# Study on polyethylene-based carbon fibers obtained by sulfonation under hydrostatic pressure

**DOI:** 10.1038/s41598-021-97529-4

**Published:** 2021-09-09

**Authors:** Jong Hyun Eun, Joon Seok Lee

**Affiliations:** Department of Fiber System Engineering, Yeungnam University, Gyeongsan, 712-749 Republic of Korea

**Keywords:** Engineering, Materials science

## Abstract

Polyethylene based carbon fibers were studied using high density polyethylene(HDPE) fibers and linear low density polyethylene(LLDPE) fibers with various melt flow index. The draw ratio of the polyethylene fibers and the sulfonation mechanism were investigated under hydrostatic pressures of 1 and 5 bar in the first time. The influence of the melt flow index of polyethylene and types of polyethylene fibers on the sulfonation reaction was studied. Carbon fibers were prepared through the sulfonation of LLDPE fibers possessing side chains with a high melt flow index. The polyethylene fibers, which exhibited thermoplastic properties and plastic behavior, were cross-linked through the sulfonation process. Their thermal properties and mechanical properties changed to thermoset properties and elastic behavior. Although sulfonation was performed under a hydrostatic pressure of 5 bar, it was difficult to convert the highly oriented polyethylene fibers because of their high crystallinity, but partially oriented polyethylene fibers could be converted to carbon fibers. Therefore, the effect of fiber orientation on fiber crosslinking, which has not been reported in previous literature, has been studied in detail, and a new method of hydrostatic pressure sulfonation has been successful in thermally stabilizing polyethylene fiber. Hydrostatic sulfonation was performed using partially oriented LLDPE fibers with a melt flow index of 20 at 130 °C for 2.5 h under a hydrostatic pressure of 5 bar. The resulting fibers were carbonized under the following conditions: 1000 °C, 5 °C/min, and five minutes. Carbon fibers with a tensile strength of 2.03 GPa, a tensile modulus of 143.63 GPa, and an elongation at break of 1.42% were prepared.

## Introduction

Carbon fibers are excellent materials for structural reinforcement in light-weight composites because of their high tensile strength, modulus, high thermal resistance, good chemical stabilities, electrical conductivities, and excellent creep resistance. On the other hand, the high production cost of carbon fibers limits their widespread use in the aerospace industry, military industry, high-performance automobile industry, or other special applications not related to price. Therefore, lowering the production cost of the carbon fibers is an important issue for expanding their use from the special applications field to the general application field. Among carbon fiber production processes, the cost of precursor fiber production contributes more than approximately 50% of the total carbon fiber production cost. Therefore, reducing the cost of these precursor fibers is essential^1–6^. Various materials have been studied to lower the cost of precursor fiber of carbon fibers, such as polyethylene, lignin, textile polyacrylonitrile, and melt spinnable polyacrylonitrile. Among these materials, polyethylene has attracted considerable attention owing to its good mechanical performance, high carbon content (86%), and ability to be melt-spun at high production rates, easily available, relatively low cost (< $1/lb), easily deformable property, and high carbon yield when converted to carbon fiber^7–13^. The price of such polyethylene is about 3 ~ 6 times cheaper than polyacrylonitrile ($3 ~ 6/ lb), which is most commonly used as a carbon fiber precursor. Polyethylene is largely classified into the following: ultra-high molecular weight (UHMWPE), which has extremely long chains of polyethylene that align in the same direction; high-density polyethylene (HDPE), which has short branches with a polymer backbone; linear low-density polyethylene (LLDPE), which is a substantially linear polymer with many short branches; and low-density polyethylene (LDPE), which has a large number of branches (approximately 2% of carbon atoms) with a polymer backbone depending on the manufacturing process. These polyethylene materials exhibit several different characteristics, such as a molecular structure, melt flow index, molecular weight, melting temperature, rheological properties, and mechanical properties^14–18^.

Several studies have examined polyethylene-based carbon fibers and various attempts to improve the tensile strength of polyethylene-based carbon fibers have been made^19–28^. J. Kim and J. Lee examined polyethylene-derived carbon fibers using LLDPE. Sulfonation was performed using sulfuric acid over a temperature range of 130 ∼ 160 °C for 1 ∼ 4 h, followed by carbonization at 950 °C for five minutes^29^. B. Barton et al. examined the high modulus low-cost carbon fibers from LLDPE^30^. S. Lee et al. suggested an effective method for polyethylene-derived carbon fibers using an electron beam irradiation process. A melt-spun linear low-density polyethylene fiber was pre-treated with an electron beam under various conditions at an intensity of 500 kGy ∼ 1500 kGy. Ninety minutes of sulfonation at 95 °C were sufficient for the 1500 kGy irradiated polyethylene fiber to produce carbon fibers with sound mechanical properties^31^.

Although carbon fibers were manufactured successfully using various polyethylene materials, such as UHMWPE, HDPE, LLDPE, and LDPE, there is no literature discussing the various types of polyethylene precursor fibers such as high density polyethylene fiber and linear low density polyethylene fiber with different melting index. Table S1 summarizes the preparation conditions of the polyethylene based carbon fiber and main characteristics of the each process. As shown in Table S1, it can be seen that the tensile strength of the polyethylene based carbon fiber studied so far is 2.1 ~ 2.4 GPa, but some literatures do not deal with the preparation conditions for each process in detail and although various types of PE were discussed, such as HDPE and LLDPE, there was no literature discussing the characteristics of two or more materials at the same time. Also, there were no literature discussing the relationship between orientation and crosslink of the precursor fiber under hydrostatic pressure condition.

Therefore, in this study, carbon fibers were manufactured by selecting HDPE and LLDPE relatively inexpensive materials. HDPE and LLDPE precursor fibers with various melt flow index and draw ratio were cross-linked through the sulfonation process. The relationship between fiber orientation and the sulfonation effect under hydrostatic pressure condition was studied, and polyethylene-based carbon fibers were successfully prepared.

## Experimental

### Materials

Polyethylene fibers were prepared using HDPE pellets (LG Chem., LOTTE Chem., Korea) and LLDPE pellets (LOTTE Chem., Korea). Table S2 lists the specifications of the HDPE pellets and LLDPE pellets. Sulfuric acid 98% (Sigma Aldrich, USA) was used as a cross-linking agent.

### Preparation of the polyethylene precursor fiber

HDPE precursor fibers and LLDPE polyethylene precursor fibers were melt-spun using a 1.0 mm diameter single nozzle extruder (Koen21 Co., Korea). Table S3 lists the detailed spinning conditions of the polyethylene precursor fibers and drawing conditions of the polyethylene precursor fibers. The partially drawn HDPE fiber and partially drawn LLDPE fiber stretched well as the melt flow index value increased; hence, a drawing process was performed at different draw ratios for each material. In the case of the fiber drawn by 33 to 40 times higher than the spinning speed, the elongation at break values showed almost 96 to 136%, which was named partially drawn fiber. In the cased of fibers drawn 1300 to 2000 times higher than the spinning speed, he elongation at break values showed around 10%, which was named fully drawn fiber.

### Sulfonation and carbonization of the polyethylene fiber

Precursor fibers, HDPE and LLDPE, were cross-linked by sulfonation using the following procedure. Sulfonation was performed using self-designed sulfonation equipment, which could control the temperature and pressure up to 1∼5 bar. The single filament fibers, such as HDPE and LLDPE fibers, were wound 50 times on a sulfonation equipment hanger to obtain a multi-filament fiber. The number of filaments in the multi-filaments was 100, and the length was 500 mm. A Teflon weight was hung at the end of the multi-filament to give a tension of 4 MPa. The prepared samples were immersed in the sulfonation equipment with 98% sulfuric acid. After immersing the specimens in the sulfuric acid, pre-treatment was performed at 80 °C for four hours. Sulfonation was carried out by varying the sulfonation temperature and pressure conditions of the pre-treated specimens. The sulfonation pressure conditions were performed under pressures of 1 bar and 5 bar, and the sulfonation temperature conditions were a temperature of 130 °C and a heating rate of 2 °C/min. In the case of HDPE fibers, such as partially drawn and fully drawn fibers, sulfonation was performed for 2, 3.5, and 5 h. For the LLDPE fibers, such as partially drawn and fully drawn fibers, sulfonation was performed for 2, 2.5, and 5 h. In the case of LLDPE fibers, because the cross-link reaction was faster than for the HDPE fibers, the specimens treated with 2.5 h sulfonation were investigated. In the case of HDPE fibers, the fibers treated with 3.5 h sulfonation were analyzed. After sulfonation, the specimens were washed with distilled water for 30 min. The washed specimens were then dried in a drying oven at 60 °C for two hours.

**Figure 1:**
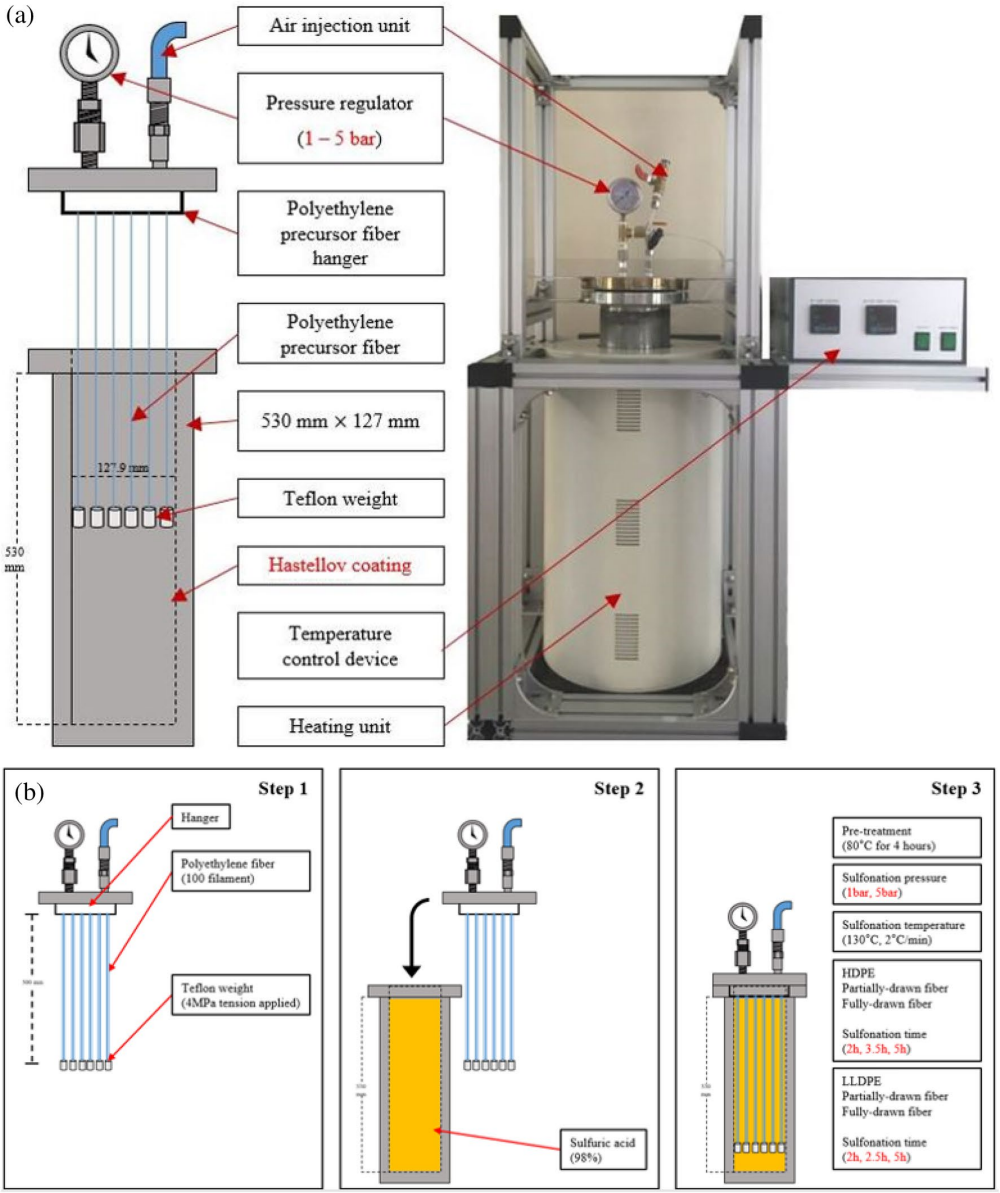
Schematic diagram of the sulfonation equipment and hydrostatic sulfonation process (**a**) A self-designed sulfonation equipment, (**b**) Schematic diagram of the hydrostatic sulfonation process.

**Figure 2:**
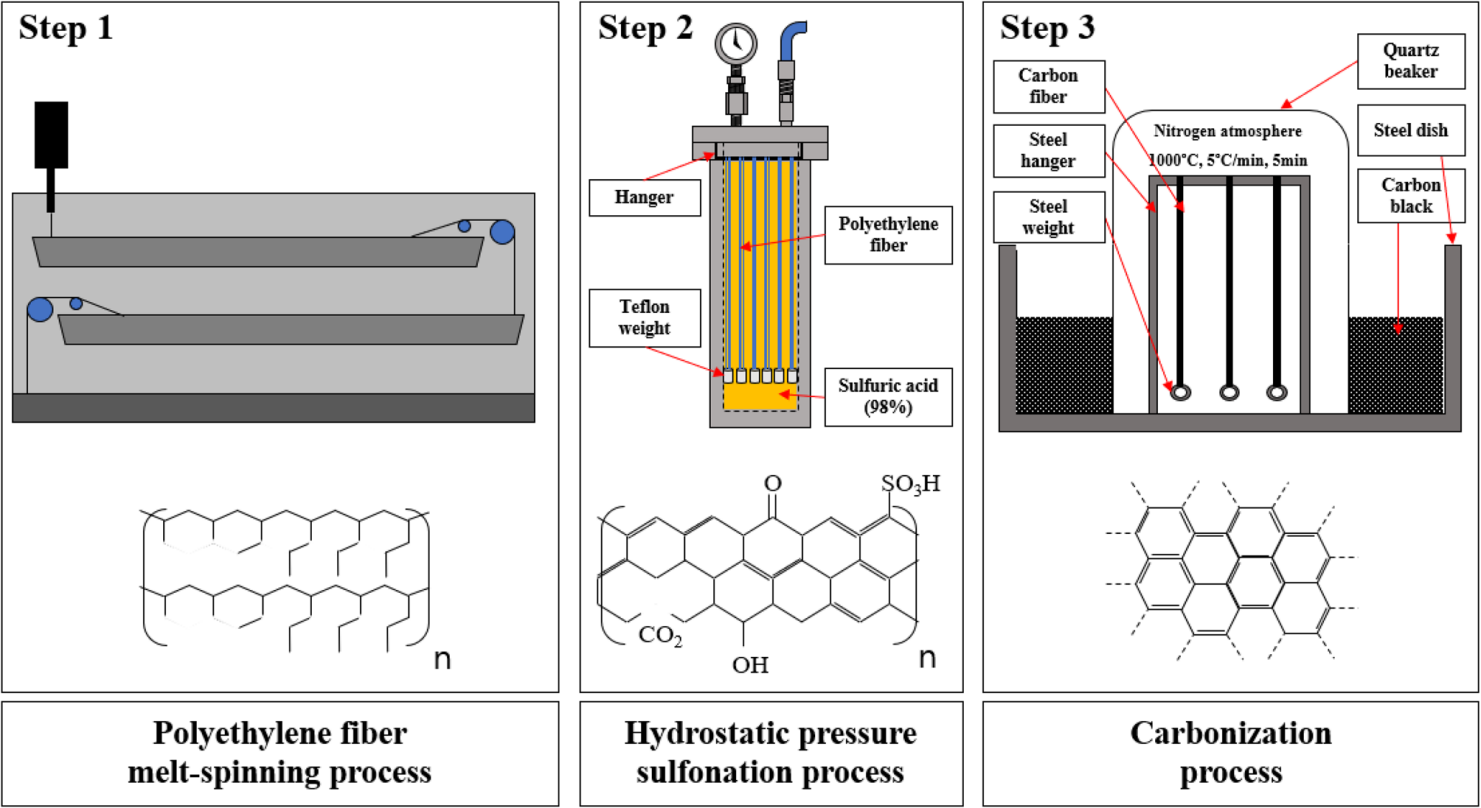
Polyethylene based carbon fiber flow chart and chemical transformation.

After sulfonation process, sulfonated polyethylene fibers were hung on a steel hanger for carbonization. The steel weight was hung at the end of the sulfonated polyethylene fiber to give a tension of 4 MPa. The prepared specimens were sealed with a quartz beaker and carbonized in a furnace (JSMF-30H, JSR, Korea) in an N_2_ atmosphere. The temperature was 1000 °C for five minutes with a heating rate of 5 °C/min. Heating was stopped after reaching the target temperature. Figure 1 presents a sulfonation equipment (Yes solutions Co., Korea) and hydrostatic sulfonation process. Figure S1 shows photographs of the before and after carbonization process. Also, Fig. 2 shows the flow chart of the preparation process of polyethylene based carbon fiber.

### Specimen code

Table S4 list the specimen code of the polyethylene precursor fibers, sulfonated polyethylene fibers and carbonized polyethylene fibers. Carbon fibers were impossible to manufacture from fully drawn polyethylene precursor fibers, whereas they were prepared successfully using partially drawn polyethylene precursor fibers.

## Results and discussion

### Polyethylene precursor fiber

#### Mechanical properties of the polyethylene precursor fiber

**Figure 3:**
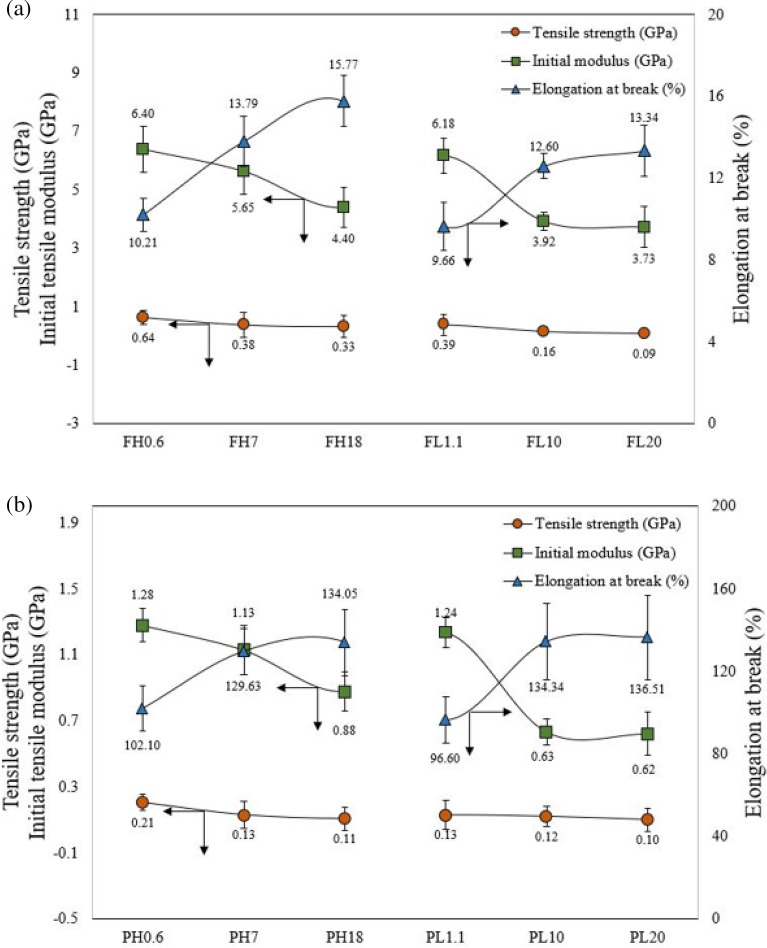
Tensile strength, initial tensile modulus, and elongation at break values of polyethylene precursor fiber with different draw ratio (**a**) Fully drawn polyethylene precursor fibers (**b**) Partially drawn polyethylene precursor fibers.

The tensile strength and initial modulus values of the polyethylene precursor fiber decreased with increasing melting flow index of the polyethylene polymer, but the elongation at break of the polyethylene precursor fiber increased (Fig. 3). This is because of the difference in molecular weight of the polyethylene polymer (Figure S2). Table S5 and S6 summarizes the tensile strength, initial tensile modulus, and elongation at break values of fully drawn polyethylene precursor fiber and partially drawn polyethylene precursor fiber with different draw ratio.

### Sulfonated polyethylene fiber

#### Morphological analysis of the sulfonated polyethylene fiber

**Figure 4:**
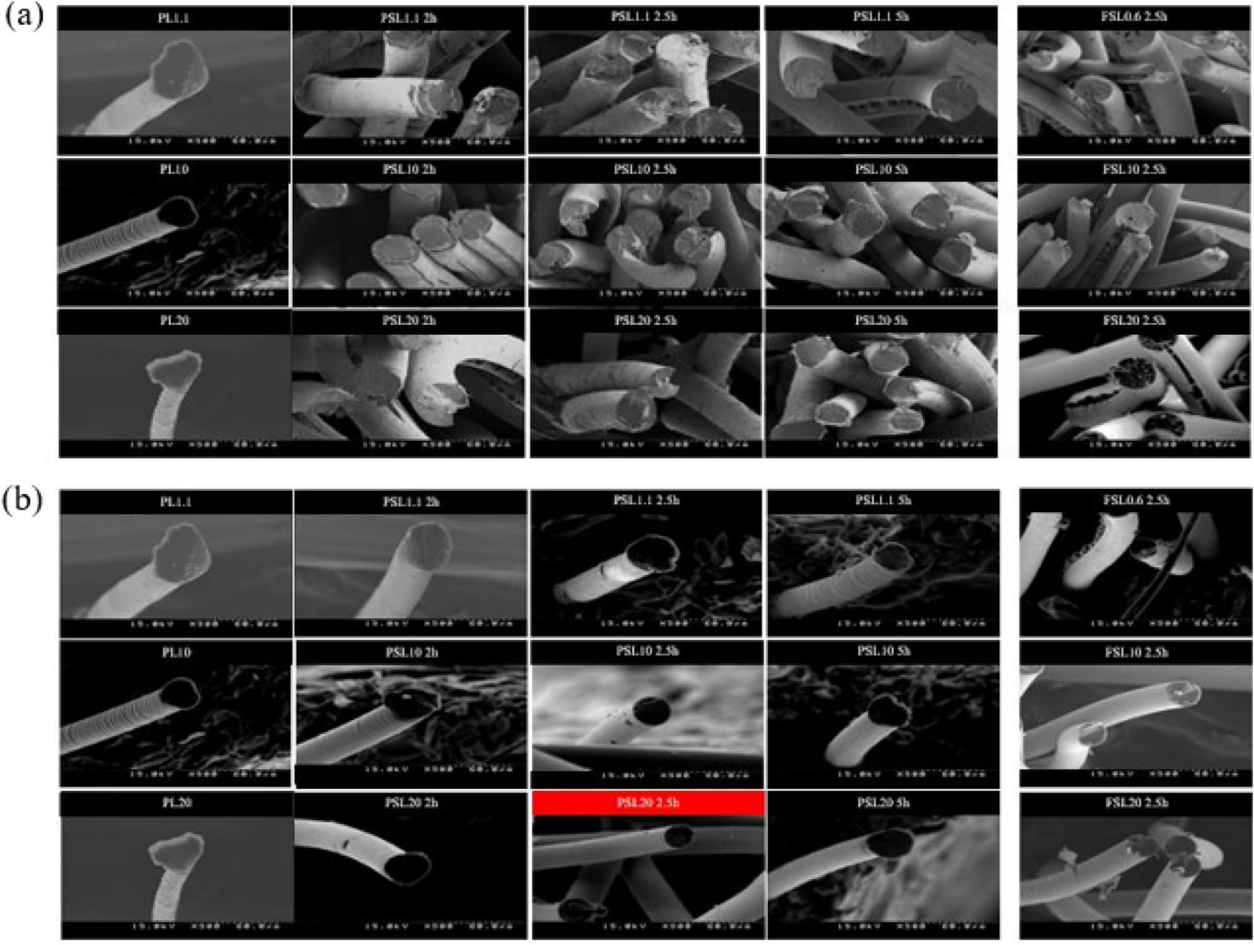
SEM images of the cross-sectional surface of the LLDPE fibers with a melt flow index of 1.1, 10, and 20 according to the sulfonation time under a pressure of 1 bar, 5 bar and temperature of 130 °C (**a**) Sulfonated fiber under 1 bar pressure, (**b**) Sulfonated fiber under 5 bar pressure.

Figure 4 shows the cross-section SEM images of the sulfonated LLDPE fibers. Figure 4a presents cross-sectional surface SEM images of the LLDPE fibers with a melt flow index of 1.1, 10, and 20 according to the sulfonation time under a pressure of 1 bar and temperature of 130 °C.

After the sulfonation treatment, the diameter of all linear density polyethylene fibers increased gradually with increasing sulfonation time. This increase in fiber diameter was attributed to the shrinkage of the longitudinal direction of the linear density polyethylene fiber due to exposure to sulfuric acid during the 130 °C sulfonation process. The LLDPE fibers also formed cracks through the fiber axial direction under all sulfonation time conditions. As the LLDPE fibers cross-link through the sulfonation process, the failure mechanism changes gradually from plastic deformation to elastic deformation. This is because the –SO_3_ crosslinks the CH_2_ chains of the LLDPE fiber; hence, atomic bonding dominates the molecule instead of intermolecular forces. In this process, residual stress remains in the fiber when shrinkage occurs in the fiber axial direction. Therefore, the fibers with elastic properties do not maintain their original shape and expand, resulting in the formation of cracks. The LLDPE fiber specimens showed a different tendency when sulfonation was performed under a high pressure of 5 bar. Figure 4b shows the cross-section SEM images of the LLDPE fibers sulfonated under a pressure of 5 bar and temperature of 130 °C. After the sulfonation treatment, the diameters of the LLDPE fiber specimens increased slightly with increasing sulfonation time, but there was almost no change in diameter compared to the HDPE fibers, and the sulfonated fibers showed a clean cross-sectional surface. This is because a high hydrostatic pressure prevents fiber shrinkage. In addition, although the partially drawn polyethylene fiber was treated for a long time, such as 5 h, a neat cross-sectional surface without a core structure was formed, and no cracks were observed through the fiber axial direction. Hence, as shown in Figure S3 the diffusion rate of the sulfuric acid into the HDPE fiber is faster under a 5 bar hydrostatic pressure than at 1 bar, so that the residual stress is reduced. In the case of fully drawn LLDPE fiber, phenomena, such as cracks in the fiber axial direction, were more prominent than that of the partially drawn LLDPE fiber. In the case of the fully-drawn LLDPE fibers, due to highly oriented the crystalline region, it is difficult for sulfuric acid to diffuse into the inside of the fiber. As a result, among all specimens including HDPE fibers and LLDPE fibers, the PSL20 specimen sulfonated for 2.5 h under 5 bar pressure was sulfonated without cracks or a core structure. Table S7, S8 lists the changes in diameter and length of the HDPE fibers and LLDPE fibers.

#### Mechanical properties of the sulfonated polyethylene fiber

Figure S4a–c present the tensile strength, initial tensile modulus, and elongation at break of the HDPE0.6, HDPE7, and HDPE18 fibers according to the sulfonation time under a pressure of 5 bar and 130 °C.

Figure S4a shows the tensile strength, initial tensile modulus, and elongation at break of a sulfonated HDPE fiber with a melt flow index of 0.6. The tensile strength of PH0.6, PSH0.6 2 h, PSH0.6 3.5 h, and PSH0.6 5 h specimens were 0.21 GPa, 0.22 GPa, 0.26 GPa, and 0.02 GPa, respectively. The tensile strength of the PSH0.6 3.5 h specimen was approximately 24% higher than the PH0.6 specimen. On the other hand, the tensile strength of the PSH0.6 5 h specimen was approximately 1200% lower than that of the PSH0.6 3.5 h specimen. The initial tensile moduli of the PH0.6, PSH0.6 2 h, and PSH0.6 3.5 h specimens were 0.58 GPa, 0.64 GPa, and 2.75 GPa, respectively. The initial tensile modulus of the PSH0.6 3.5 h specimen was approximately 374% higher than that of the PH0.6 specimen. The elongation at break of the PH0.6, PSH0.6 2 h, and PSH0.6 3.5 h specimens were 102.10%, 39.06%, and 6.40%, respectively. The elongation at break of the PSH0.6 3.5 h specimen was approximately 1495% higher than that of the PH0.6 specimen. On the other hand, the tensile strength, initial tensile modulus, and elongation at break of the FSH0.6 3.5 h specimen were 0.01 GPa, 1.85 GPa, and 10.58%, respectively. The tensile strength and initial tensile modulus of the FSH0.6 3.5 h specimen were approximately 2500% and 48% lower, respectively, than that of the PSH0.6 3.5 h specimen, respectively. The elongation at break value of the FSH0.6 3.5 h specimen was 64% higher than that of the PSH0.6 3.5 h specimen.

Figure S4b shows the tensile strength, initial tensile modulus, and elongation at break of HDPE7 fiber. The tensile strength of the PH7, PSH7 2 h, PSH7 3.5 h, and PSH7 5 h specimens were 0.13 GPa, 0.16 GPa, 0.18 GPa, and 0.02 GPa, respectively. The tensile strength of the PSH7 3.5 h specimen was approximately 38% higher than that of the PH7 specimen. On the other hand, the tensile strength of the PSH7 5 h specimen was approximately 800% lower than that of the PSH7 3.5 h specimen. The initial tensile moduli of the PH7, PSH7 2 h, and PSH7 3.5 h specimens were 1.13 GPa, 2.74 GPa, and 5.36 GPa, respectively. The initial tensile modulus of the PSH7 3.5 h specimen was approximately 374% lower than that of the PH7 specimens. The elongation at break of the PH7, PSH7 2 h, and PSH7 3.5 h specimens were 129.63%, 32.54%, and 3.50%, respectively. The elongation at break of the PSH7 3.5 h specimen was approximately 3604% lower than that of the PH7 specimen. The tensile strength, initial tensile modulus, and elongation at break of the FSH7 3.5 h specimen were 0.09 GPa, 3.21 GPa, and 12.90%, respectively. The tensile strength and initial tensile modulus of the FSH7 3.5 h specimen was approximately 100% and 66% lower, respectively, than the PSH7 3.5 h specimen. The elongation at break of the FSH7 3.5 h specimen was 268% higher than that of the PSH7 3.5 h specimen.

Figure S4c shows the tensile strength, initial tensile modulus, and elongation at break of the HDPE18 fiber. The tensile strength of the PH18, PSH18 2 h, PSH18 3.5 h, and PSH18 5 h specimens were 0.11 GPa, 0.17 GPa, 0.19 GPa, and 0.08 GPa, respectively. The tensile strength of the PSH18 3.5 h specimen was approximately 72% lower than that of the PH18 specimen. On the other hand, the tensile strength of the PSH18 5 h specimen was approximately 137% lower than that of the PSH18 3.5 h specimen. The initial tensile moduli of the PH18, PSH18 2 h, and PSH18 3.5 h specimens were 0.88 GPa, 2.98 GPa, and 5.87 GPa, respectively. The initial tensile modulus of the PSH18 3.5 h specimen was approximately 567% higher than that of the PH18 specimens. The elongation at break of the PH18, PSH18 2 h, and PSH18 3.5 h specimens were 134.05%, 22.12%, and 3.25%, respectively. The elongation at break of the PSH18 3.5 h specimen was approximately 4025% lower than that of the PH18 specimen. The tensile strength, initial tensile modulus, and elongation at break of the FSH18 3.5 h specimen were 0.03 GPa, 3.21 GPa, and 38.57%, respectively. The tensile strength and initial tensile modulus of the FSH18 3.5 h specimen were approximately 533% and 83% lower, respectively, than that of the PSH18 3.5 h specimen. The elongation at break of the FSH18 3.5 h specimen was 348% higher than that of the PSH18 3.5 h specimen.

In the case of partially drawn HDPE fiber, the tensile strength and initial tensile modulus values tended to increase with increasing sulfonation time, regardless of the melt flow index, but the elongation at break decreased rapidly. Hence, the mechanical behavior of the HDPE changed from plastic behavior to elastic behavior. This is because the properties of the fibers change elastically in that HDPE, an aliphatic organic compound, is converted to an aromatic organic compound by SO_3_ from sulfuric acid, and a benzene ring is formed. This decreases the molecular chain slippage and enhances the cross-linking of polyethylene molecules. The bonding force between atoms is dominant compared to van der Waals and intermolecular attractions while forming a benzene ring. In addition, when sulfonation was performed for five hours, the tensile strength of the partially drawn HDPE fibers decreased dramatically. The fiber was so brittle that it was impossible to measure the strength using the universal testing machine. The diffusion of sulfuric acid occurs preferentially in the amorphous region of the partially drawn HDPE fiber, which has no branches in the polymer backbone. The sulfuric acid then attacks the crystalline region of the polymer and damages the fiber.

Interestingly, in the case of the fully drawn HDPE fiber specimen, the tensile strength decreased rapidly. There is a relationship between the increase in crystallinity due to the high orientation of the HDPE fiber and sulfuric acid penetration. Fibers with high crystallinity are difficult to penetrate by sulfuric acid. Moreover, the axial cracks caused by the residual stress inside the fiber generated during the sulfonation process reduce the mechanical properties of the sulfonated HDPE fiber.

**Figure 5:**
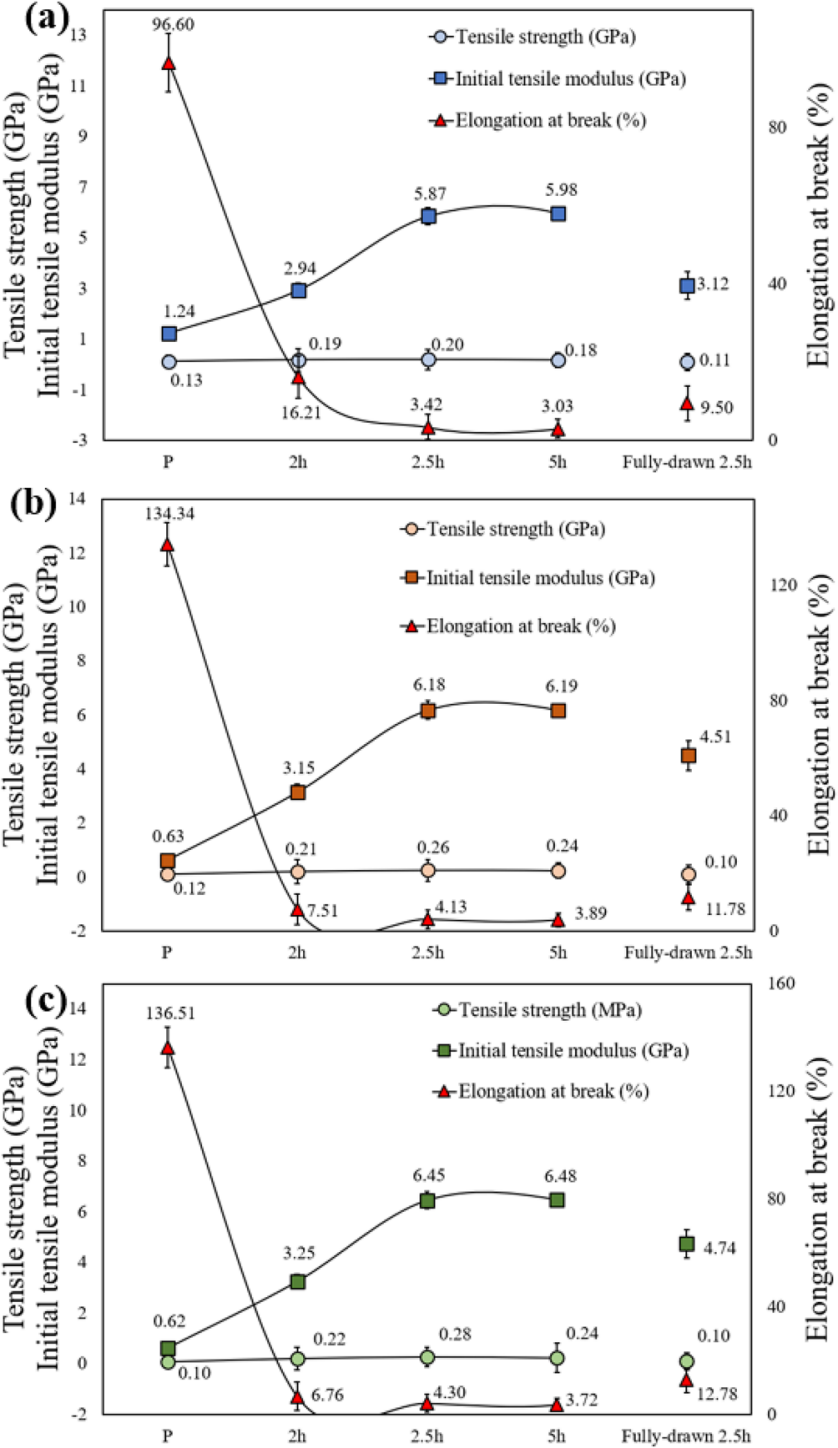
Tensile strength, initial tensile modulus, and elongation at break of the sulfonated LLDPE with various sulfonation time under a pressure of 5 bar and temperature of 130 °C (**a**) Sulfonated LLDPE fiber with melt flow index of 1.1, (**b**) Sulfonated LLDPE fiber with melt flow index of 10, (**c**) Sulfonated LLDPE fiber with melt flow index of 20.

Figure 5a–c show the tensile strength, initial tensile modulus, and elongation at break of the LLDPE1.1, LLDPE10, and LLDPE20 fibers according to the sulfonation time under a pressure of 5 bar and temperature of 130 °C.

Figure 5a shows the tensile strength, initial tensile modulus, and elongation at break of the sulfonated LLDPE fibers with a melt flow index of 1.1. The tensile strengths of the PL1.1, PSL1.1 2 h, PSL1.1 2.5 h, and PSL1.1 5 h specimens were 0.13 GPa, 0.19 GPa, 0.20 GPa, and 0.18 GPa, respectively. The tensile strength of the PSL1.1 2.5 h specimen was approximately 53% higher than that of the PL1.1 specimen. On the other hand, the tensile strength of the PSL1.1 5 h specimen was approximately 11% lower than that of the PSL1.1 2.5 h specimen.

The initial tensile moduli of the PL1.1, PSL1.1 2 h, PSL1.1 2.5 h, and PSL1.1 5 h specimens were 1.24 GPa, 2.94 GPa, 5.87 GPa, and 5.98 GPa, respectively. The initial tensile modulus of the PSL1.1 2.5 h specimen was approximately 373% higher than that of the PL1.1 specimen. The elongation at break of the PL1.1, PSL1.1 2 h, PSL1.1 2.5 h, and PSL1.1 5 h specimens were 96.60%, 16.21%, 3.42%, and 3.03%, respectively. The elongation at break of the PSL1.1 2.5 h specimen was approximately 2724% lower than that of the PL1.1 specimen. The tensile strength, initial tensile modulus, and elongation at break of FSL1.1 2.5 h were 0.11 GPa, 3.12 GPa, and 9.50%, respectively. The tensile strength and initial tensile modulus of the FSL1.1 2.5 h specimen were approximately 81% and 88% lower, respectively, than those of the PSL1.1 2.5 h specimen. The elongation at break of the FSL1.1 2.5 h was 177% higher than that of the PSL1.1 2.5 h specimen.

Figure 5b presents the tensile strength, initial tensile modulus, and elongation at break of the sulfonated LLDPE fibers with a melt flow index of 10. The tensile strength of the PL10, PSL10 2 h, PSL10 2.5 h, and PSL10 5 h specimens were 0.12 GPa, 0.21 GPa, 0.26 GPa, and 0.24 GPa, respectively. The tensile strength of the PSL10 2.5 h specimen was approximately 117% higher than that of the PL10 specimen. On the other hand, the tensile strength of the PSL10 5 h specimen was approximately 8% lower than that of the PSL10 2.5 h specimen. The initial tensile moduli of the PL10, PSL10 2 h, PSL10 2.5 h, and PSL10 5 h specimens were 0.63 GPa, 3.15 GPa, 6.18 GPa, and 6.19 GPa, respectively. The initial tensile modulus of the PSL10 2.5 h specimen was approximately 881% higher than that of the PL10 specimen. The elongation at break of the PL10, PSL10 2 h, PSL10 2.5 h, and PSL10 5 h specimens were 134.34%, 7.51%, 4.13%, and 3.89%, respectively. The elongation at break of the PSL10 2.5 h specimen was approximately 3153% lower than that of the PL10 specimen. The tensile strength, initial tensile modulus, and elongation at break of FSL10 2.5 h were 0.10 GPa, 4.51 GPa, and 11.78%, respectively. The tensile strength and initial tensile modulus of the FSL10 2.5 h specimen were approximately 160% and 37% lower, respectively, than those of the PSL10 2.5 h specimen. The elongation at break value of the FSL10 2.5 h specimen was 185% higher than that of the PSL10 2.5 h specimen.

Figure 5c shows the tensile strength, initial tensile modulus, and elongation at break of the sulfonated LLDPE fibers with a melt flow index of 20. The tensile strength of the PL20, PSL20 2 h, PSL20 2.5 h, and PSL20 5 h specimens were 0.10 GPa, 0.22 GPa, 0.28 GPa, and 0.24 GPa, respectively. The tensile strength of the PSL20 2.5 h specimen was approximately 180% higher than that of the PL20 specimen. In contrast, the tensile strength of the PSL20 5 h specimen was approximately 16% lower than that of the PSL20 2.5 h specimen. The initial tensile moduli of the PL20, PSL20 2 h, PSL20 2.5 h, and PSL20 5 h specimens were 0.62 GPa, 3.25 GPa, 6.45 GPa, and 6.48 GPa, respectively. The initial tensile modulus of the PSL20 2.5 h specimen was approximately 940% higher than that of the PL20 specimen. The elongation at break of the PL20, PSL20 2 h, PSL20 2.5 h, and PSL20 5 h specimens were 136.51%, 6.76%, 4.30%, and 3.72%, respectively. The elongation at break of the PSL20 2.5 h specimen was approximately 3075% lower than that of the PL20 specimen.

In contrast, the tensile strength, initial modulus, and elongation at break of the FSL20 2.5 h specimen were 0.10 GPa, 4.74 GPa, and 12.78%, respectively. The tensile strength and initial tensile modulus of the FSL20 2.5 h specimen were approximately 160% and 37% lower, respectively, than those of the PSL20 2.5 h specimen. The elongation at break of the FSL20 2.5 h specimen was 197% higher than that of the PSL20 2.5 h specimen.

In the case of partially drawn LLDPE fiber with a melt flow index of 1.1, 10, and 20, the tensile strength and initial tensile modulus values tended to increase with increasing sulfonation time, but the elongation at break decreased dramatically. The changes in mechanical properties showed a similar tendency to those of the partially drawn HDPE sulfonated fibers. On the other hand, the initial tensile modulus of sulfonated partially drawn HDPE fibers was significantly higher because of the molecular structure of the LLDPE fiber. LLDPE exhibits a molecular structure with many branches in the main polymer backbone. Moreover, the cross-linking efficiency was increased dramatically by these branches. Therefore, most of the aliphatic components, such as CH_2_ and CH_3_, which constitute the main polymer backbone and side chains (branches) of the LLDPE fiber, participated in the reaction with sulfuric acid to form a benzene ring. Thus, the initial tensile modulus of the LLDPE sulfonated fiber was relatively high.

Despite the long sulfonation process, such as five hours, the mechanical properties of the partially drawn LLDPE sulfonated fibers did not significantly decrease compared to the partially drawn HDPE sulfonated fibers. This is because the sulfonated LLDPE fiber forms a benzene ring, and the molecular structure is arranged regularly. Hence, damage to the polymer structure by sulfuric acid does not occur. In addition, the tensile strength of the fully drawn LLDPE fiber specimens decreased rapidly. Hence, there is a relationship between the increase in crystallinity due to the high orientation of the LLDPE fiber and the penetration of sulfuric acid into the fiber. Fibers with high crystallinity are difficult to penetrate by sulfuric acid. Moreover, axial cracks formed by the residual stress inside the fiber generated during the sulfonation process decreases the mechanical properties of the sulfonated LLDPE fiber^26,29^.

**Figure 6:**
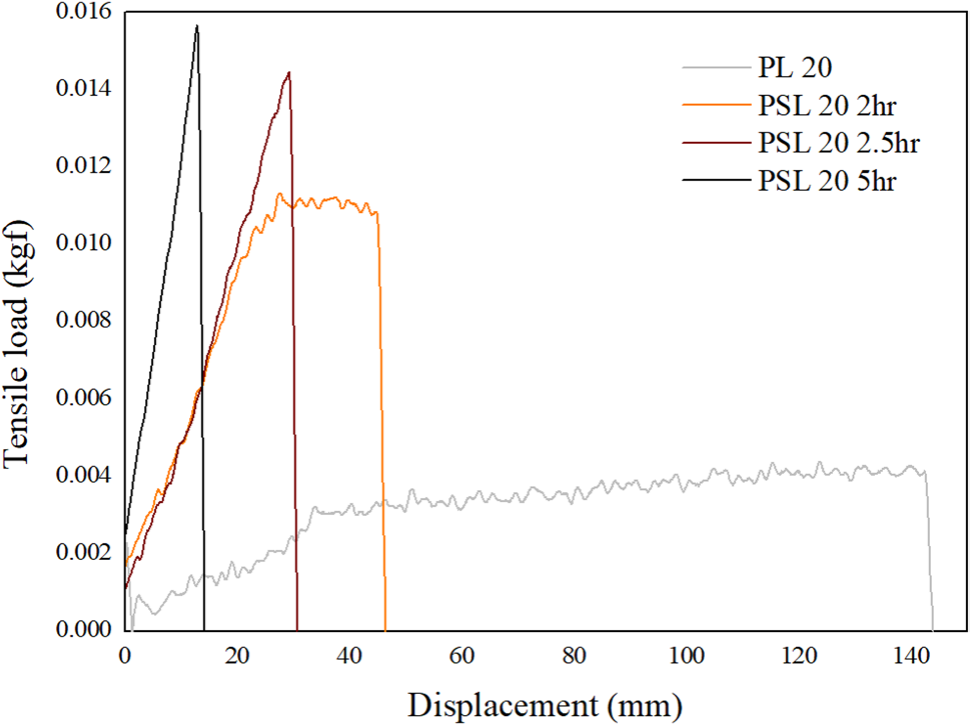
Representative load–displacement curves of the LLDPE fiber (Melt flow index 20) and sulfonated LLDPE fiber (Melt flow index 20) with various sulfonation time under a pressure of 5 bar and temperature of 130 °C.

A load–displacement curve was prepared to verify in more detail the change in the properties of polyethylene fibers according to the sulfonation process. Figure 6 shows a representative tensile load–displacement curve according to various sulfonation time under 5 bar pressure of LLDPE fiber with a melt flow index of 20. In the case of PL20, the load–displacement curve of a typical plastic behavior material was shown. It can be confirmed that reversible elastic behavior is shown until the displacement value of around 30 mm, however irreversible plastic behavior, that is, permanent deformation, occurs at displacement value of around 30 mm. In the case of the LLDPE fiber sulfonated for 2 h, the tensile modulus was significantly increased and the permanent irreversible plastic region was decreased enormously. This phenomenon means that intermolecular slippage due to crosslinking is significantly reduced. In the case of LLDPE fibers treated with sulfuric acid for 2.5 and 5 h, the load–displacement curve of a typical elastic behavior material was shown, indicating that the properties were completely transformed to reversible material.

#### Thermal properties of the sulfonated polyethylene fiber

Figure S5a shows the DSC curves of fully drawn sulfonated HDPE fiber under a sulfonation pressure of 5 bar and sulfonation temperature of 130 °C. The melting temperature of the FH0.6, FSH0.6 2 h, FSH0.6 3.5 h, and FSH0.6 5 h specimens were 137.57 °C, 136.36 °C, 132.21 °C, and 135.21 °C, respectively. The melting temperature of the FSH0.6 3.5 h specimen was approximately 4% lower than that of the FH0.6 specimen. The melting temperatures of the FH7, FSH7 2 h, FSH7 3.5 h, and FSH7 5 h specimens were 135.36 °C, 132.43 °C, 131.21 °C, and 130.21 °C, respectively. The melting temperature of the FSH7 3.5 h specimen was approximately 3% lower than that of the FH7 specimen. The melting temperatures of the FH18, FSH18 2 h, FSH18 3.5 h, and FSH18 5 h specimens were 132.57 °C, 130.29 °C, 129.41 °C, and 128.48 °C, respectively. The melting temperature of the FSH18 3.5 h specimen was approximately 2% lower than that of the FH18 specimen. Table S9 lists the melting temperatures and endothermic enthalpies of the fully drawn sulfonated HDPE fibers.

The melting temperature and endothermic enthalpy decreased gradually with increasing sulfonation time. In the case of the specimens sulfonated for 5 h, only a weak melting point peak was observed, regardless of the melt flow index of the HDPE. Moreover, the endothermic enthalpy was also very low, which means that the thermal properties of the HDPE fibers were cross-linked by SO_3_ in sulfuric acid to change from thermoplastic to thermoset properties. On the other hand, although sulfonation was performed for a long time, such as 5 h, a small endothermic enthalpy still appeared, indicating that the polymers do not participate in the crosslinking reaction and remain aliphatic organic compounds.

Figure S5b presents the DSC curve of partially drawn sulfonated HDPE fiber under a sulfonation pressure of 5 bar and sulfonation temperature of 130 °C. The melting temperature of the PH0.6, PSH0.6 2 h, and PSH0.6 3.5 h specimens were 135.21 °C, 134.1 °C, and 132.79 °C, respectively. The melting temperature of the PH0.6 3.5 h specimen was approximately 1.8% lower than that of the PH0.6 specimen. The PSH0.6 5 h specimen showed no melting point. The melting temperatures of the PH7 and PSH7 2 h specimens were 132.50 °C and 131.43 °C, respectively. The PSH7 3.5 h and PSH7 5 h specimens showed no melting point. The melting temperatures of the PH18 and PSH18 2 h specimens were 130.50 °C and 130.19 °C, respectively, but the PSH18 3.5 and PSH18 5 h specimens showed no melting point. Table S10 lists the melting temperatures and endothermic enthalpies of the fully drawn sulfonated HDPE fibers.

As the sulfonation time increased, the melting temperature and endothermic enthalpy decreased gradually. In the case of the specimens sulfonated for 3.5 h and 5 h except for PSH0.6 specimens, regardless of the melt flow index of the HDPE, the melting point peak disappeared along with the endothermic enthalpy. This means that the HDPE fibers were totally cross-linked by the SO_3_ group in sulfuric acid, resulting in a change in the thermal properties from thermoplastic to thermoset. In addition, the melting point peak disappeared relatively easily because sulfuric acid easily penetrated the amorphous region of the partially drawn HDPE fibers.

**Figure 7:**
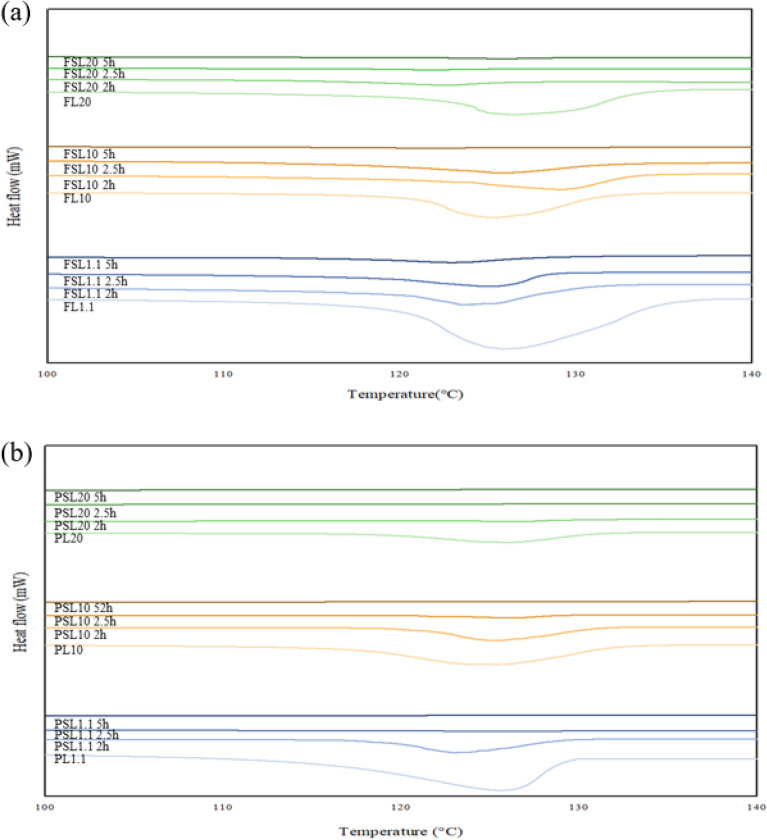
DSC curves of the LLDPE fibers with melt flow index of 1.1, 10, and 20 according to the sulfonation time under a pressure of 5 bar and temperature of 130 °C (**a**) Fully drawn sulfonated LLDPE fiber, (**b**) Partially drawn sulfonated LLDPE fiber.

Figure 7a shows the DSC curve of the fully drawn sulfonated LLDPE fiber under a sulfonation pressure of 5 bar and a sulfonation temperature of 130 °C. The melting temperatures of the FL1.1, FSL1.1 2 h, and FSL1.1 2.5 h specimens were 125.54 °C, 122.11 °C, and 121.08 °C, respectively. In the case of the FSL1.1 5 h specimen, the melting point of the LLDPE fiber was not observed. The melting temperature of the 2.5-h specimen was approximately 3.6% lower than that of the FL1.1 specimen. The melting temperatures of the FL10, FSL10 2 h, and FSL10 2.5 h specimens were 123.48 °C, 122.11 °C, and 121.11 °C, respectively. The melting point of the LLDPE fiber was not observed in the case of the FSL10 5 h specimen. The melting temperature of the 2.5-h specimen was approximately 1.9% lower than that of the FL10 specimen. The melting temperature of FL20 and FSL20 2 h were 122.67 °C and 120.03 °C, respectively. The melting point of the LLDPE fiber was not observed in the case of the FSL20 3 h and FSL20 5 h specimens. Table S11 lists the melting temperature and endothermic enthalpy of fully drawn sulfonated LLDPE fibers.

As the sulfonation time increased, the melting temperature and endothermic enthalpy decreased gradually, regardless of the melt flow index of the LLDPE fibers. In contrast to the fully drawn HDPE fiber, the FSL10 and FSL20 specimens showed complete crosslinking under the sulfonation condition of 5 h. Although the crystallinity of polyethylene was increased by the drawing process, the crosslinking reaction was promoted by the branches. In addition, for specimens without a melting point, the thermal properties of the polyethylene changed completely from thermo-plastic to thermo-set.

Figure 7b shows the DSC curve of partially drawn sulfonated LLDPE fiber under a sulfonation pressure of 5 bar and a sulfonation temperature of 130 °C. The melting temperatures of the PL1.1, PSL1.1 2 h, and PSL1.1 2.5 h specimens were 123.5 °C, 121.21 °C, and 120.08 °C, respectively. The PSL1.1 5 h specimen showed no melting point. The melting temperature of the PSL1.1 2.5 h specimen was approximately 2.8% lower than that of the PL1.1 specimen. The melting temperatures of the PL10, PSL10 2 h, and PSL10 2.5 h specimens were 122.43 °C, 121.85 °C, and 120.74 °C, respectively. The FSL10 5 h specimen showed no melting point of the LLDPE fiber. The melting temperature of the PSL10 2.5 h specimen was approximately 1.3% lower than that of the PL10 specimen. The melting temperature of the PL20 and PSL20 2 h specimens were 121.19 °C and 120.01 °C, respectively. In contrast, the PSL20 2.5 h and PSL20 5 h specimens showed no melting point compared to the PL20 specimen. Table S12 lists the melting temperatures and endothermic enthalpies of the partially drawn sulfonated LLDPE fibers.

The crosslinking efficiency of the partially drawn LLDPE fiber was superior to the fully drawn HDPE fibers, partially drawn HDPE fibers, and fully drawn LLDPE fibers. This is due to the thin fiber diameter of LLDPE, many polymer side chains inside the polymer backbone structure, and the proper distribution of amorphous regions^8,9,18^.

#### Molecular structure change of the sulfonated polyethylene fiber

Figures S6a–c show the typical ^1^H NMR spectra of the PL1.1, PL10, and PL20 specimens, respectively. The peak at 0.7–1.3 ppm was assigned to a proton of the CH3 group; the peak at 1.2–1.4 ppm corresponded to a proton of the CH_2_ group, and the peak at 1.4–1.7 ppm was attributed to a proton of the CH group. Because the LLDPE is a structure with many side chains (branches), peaks were generated for a large range of primary alkyl, secondary alkyl, and tertiary alkyl groups. On the other hand, there was no peak at 6.5–8.0 ppm corresponding to the protons of an aromatic ring. This indicates that pure LLDPE without sulfonation is also an aliphatic organic compound. In the case of HDPE precursor fibers, only two distinct peaks were observed because the HDPE fiber is the dominant structure of the main chain, and peaks of primary alkyl and secondary alkyl were generated in all HDPE samples (Figure S7).

In the case of HDPE and LLDPE fibers treated with sulfonation under a pressure of 1 bar, they were crosslinked with sulfuric acid, and a benzene peak could be observed in the range of 6.5–8.0 ppm(Figure S8 and Figure S9), and this benzene peak was remarkably observed under the sulfonation pressure of 5 bar.

**Figure 8:**
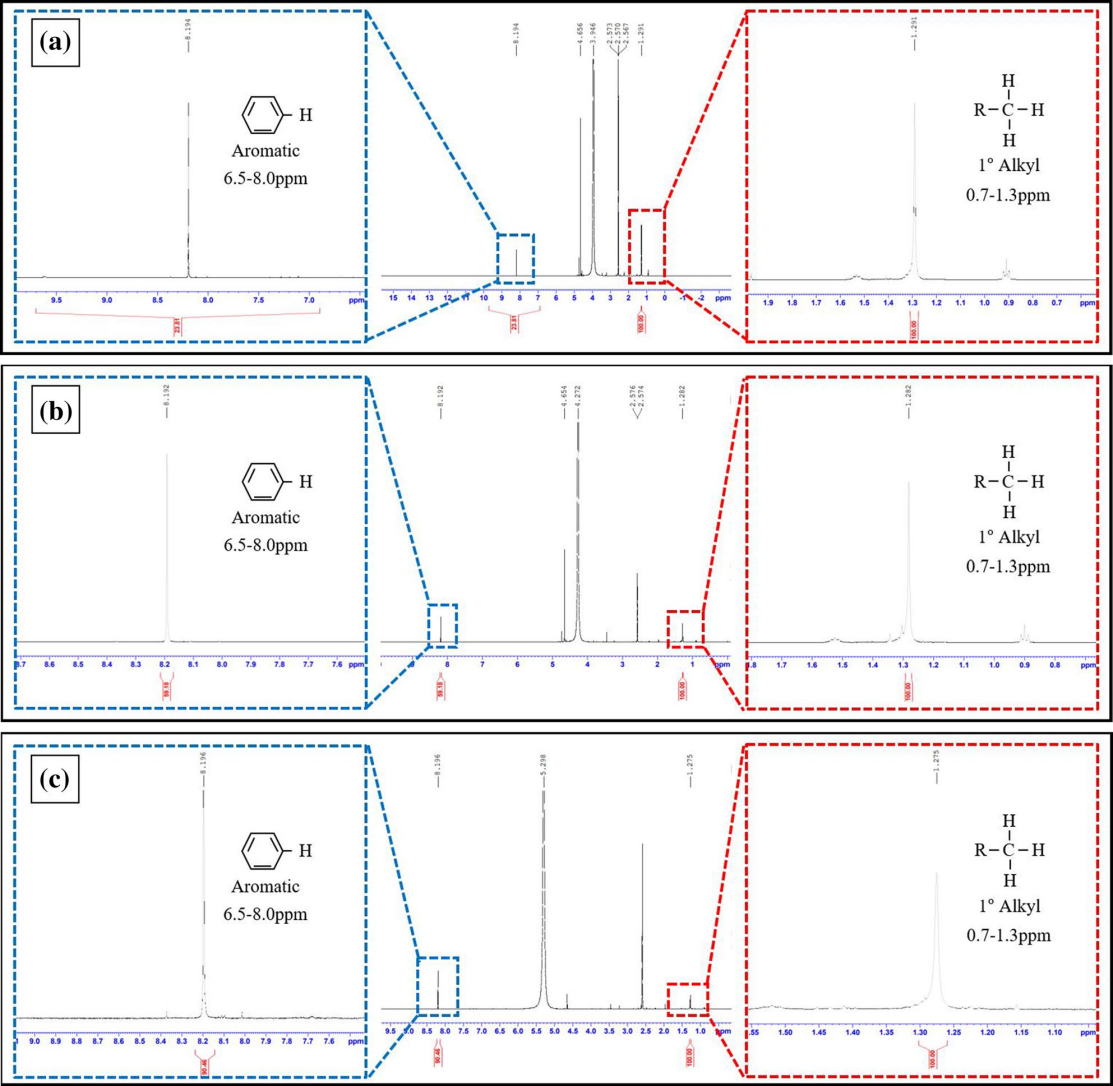
^**1**^H NMR spectrum and hydrogen peak assignments of sulfonated partially drawn LLDPE fibers under sulfonation conditions of 5 bar for 2.5 h (**a**) PSL1.1, (**b**) PSL10, (**c**) PSL20.

Figure 8a–c show the typical ^1^H NMR spectrum of the PSL1.1, PSL10, and PSL20 specimens. In the case of partially drawn sulfonated LLDPE fiber that was sulfonated at 5 bar, most of the primary alkyl groups, secondary alkyl groups, and tertiary alkyl groups disappeared, and protons for an aromatic ring were observed at 6.5–8.0 ppm. The aromatic peak dominated the molecular structure of the polymer. This result provides conclusive evidence that polyethylene fibers, which were aliphatic organic compounds, were cross-linked by sulfonation and converted to aromatic organic compounds. Aliphatic organic compounds have a relatively weak bond strength of 364 kJ/mol and are easily phase-changed and thermally decomposed by the movement of molecular chains at carbonization temperature. On the other hand, aromatic organic compounds are quite strong with a bonding strength of 518 kJ/mol, so that they are not decomposed easily even at carbonization temperature. These benzene rings are formed more easily in LLDPE materials with a high melt flow index than LLDPE with a relatively low melt flow index of 1.1. However, in the case of HDPE fiber, even though sulfonated under 5 bar pressure, it could not completely changed aromatic polymer due to the molecular structure without branches (Figure S10). Table S13, S14, and S15 listed the ^**1**^H NMR spectrum chemical shift and integration values of the polyethylene precursor fibers and sulfonated polyethylene fibers.

In order to observe the change in the molecular structure of the polyethylene fiber in more detail, FT-IR analysis was performed.

Figure S11 show the FT-IR spectra of sulfonated LLDPE fibers. The PL1.1, PL10, and PL20 specimens (Figure S11a–c) showed absorptions at 2928 cm^−1^ and 2859 cm^−1^ for the C–H asymmetric and symmetric stretching vibrations, respectively. Absorptions at 1469 cm^−1^ (C–H bending vibration) and 738 cm^−1^ (C–H bending) were detected. All of these peaks were attributed to aliphatic organic compounds in LLDPE. Specimens FSL 1.1,10, and 20 treated by sulfonation revealed aromatic C–C stretching vibrations and aromatic C-H out of plane bending peaks concomitantly CH_2_ and CH_3_ peaks at 1500–1400 cm^−1^ and 674 cm^−1^, respectively. Hence, the aliphatic organic compounds changed to aromatic organic compounds. On the other hand, in the PSL1.1, 10, and 20 specimens, the CH_2_ and CH_3_ peaks disappeared, and only aromatic C–C stretching vibrations (1500–1400 cm^−1^) and aromatic C–H out of plane bending vibrations (674 cm^−1^) were detected. Therefore, the LLDPE fibers changed from aliphatic organic compounds to aromatic organic compounds.

#### Orientation of LLDPE fibers and the molecular structure of sulfonated linear low-density fibers

LLDPE fibers with melt flow index of 20, which had superior sulfonation efficiency than HDPE fibers, were selected, and the orientation characteristics according to fiber drawing and molecular structure of the sulfonated LLDPE were analyzed qualitatively by WAXD.

**Figure 9:**
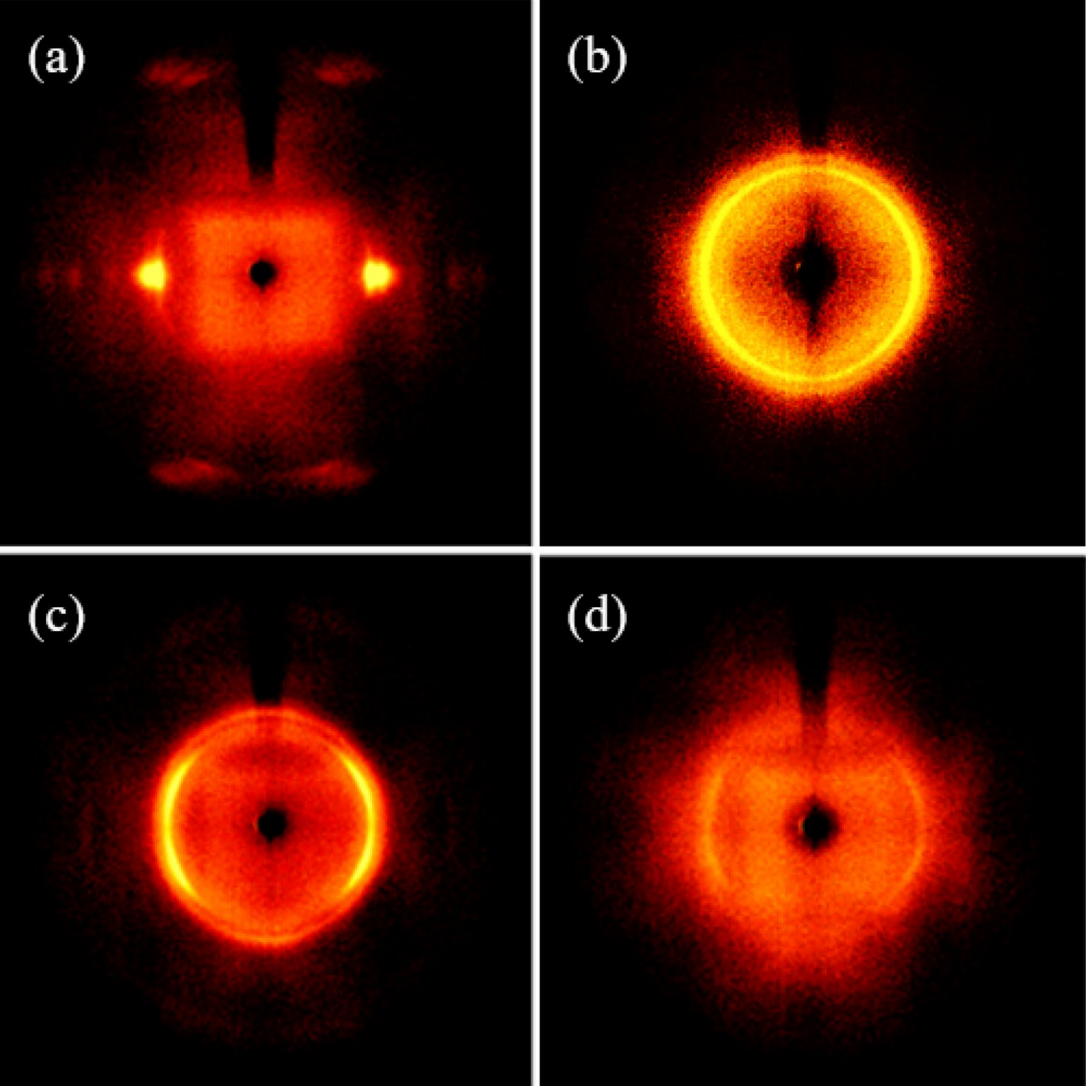
2D-WAXD pattern of LLDPE precursor fibers and sulfonated LLDPE fibers with melt flow index of 20 at a sulfonation time of 2.5 h under a pressure of 5 bar and temperature of 130 °C (**a**) FL20, (**b**) FSL20, (**c**) PL20, (**d**) PSL20.

Figure 9 presents the 2D-WAXD pattern of LLDPE precursor fibers and sulfonated LLDPE fibers with melt flow index of 20. The fully drawn LLDPE fiber (Fig. 9a) showed remarkable peaks at 2θ = 23°. The X-ray pattern of the fully drawn LLDPE fiber shows that the ring is partially bright in the equatorial direction, and a clear crystal orientation result of the LLDPE fiber appeared. This pattern indicates that the crystalline phase of the LLDPE fiber was well oriented, indicating that the polymer chain axis (c-axis) is oriented parallel to the fiber drawing direction. This means that the level of orientation is improved dramatically by the drawing process. Moreover, the crystalline phase became more oriented along the fiber axis. Figure 9b shows the 2D-WAXD pattern of FSL20 specimen. This 2D-WAXD pattern means that the crystal regions lost their orientation and were arranged randomly, indicating that crystals with melting points exist. The presence of crystals with melting points suggests that the fibers may eventually fail to maintain their shape during the carbonization step and may be pyrolyzed. Figure 9c shows the 2D-WAXD pattern of PL20 specimen. The partially drawn LLDPE fiber showed remarkable peaks at 2θ = 23°. The rings of the X-ray pattern of partially drawn LLDPE fiber appear brightly over a wider area than the fully drawn LLDPE fiber. This indicates that the LLDPE fibers were partially drawn and have relatively isotropic properties compared to the fully drawn LLDPE fibers. Figure 9d shows the 2D-WAXD pattern of PSL20 specimen. Very fuzzy rings were observed in the X-ray pattern of the sulfonated LLDPE fiber. These patterns are typically observed in fibers exhibiting isotropic properties. Moreover, it is believed that directional LLDPE fibers are cross-linked by sulfuric acid to form benzene rings. LLDPE changed to an aromatic structure and lost its orientation^30^.

### Polyethylene-based carbon fibers

#### Molecular structure change of the polyethylene based carbon fiber

**Figure 10:**
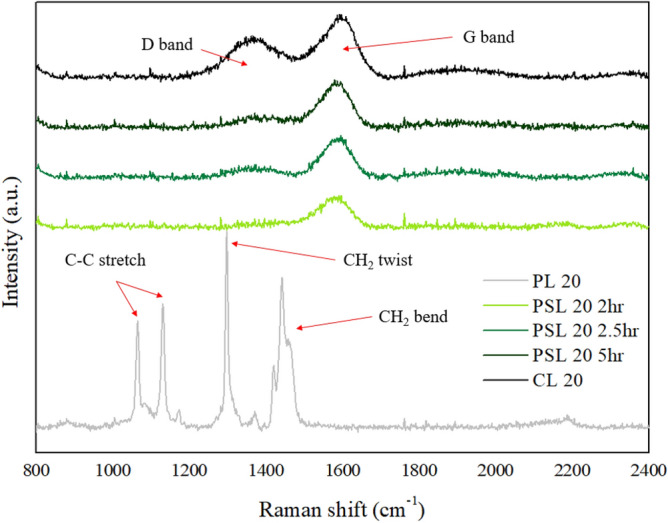
Raman spectrum of LLDPE fiber (PL20), sulfonated LLDPE fiber (PSL) treated under 5 bar and various time conditions, and polyethylene based carbon fiber prepared by LLDPE fiber.

Figure 10 shows the Raman spectrum of LLDPE fiber (PL20), sulfonated LLDPE fiber(PSL) treated under 5 bar and various time conditions, and polyethylene based carbon fiber prepared by LLDPE fiber. In the case of LLDPE fiber, it shows a typical Raman spectrum of polyethylene which is the sharp peaks in the vibrational bonds of alkyl groups C_n_H_2n+1_ : the C–C symmetric and asymmetric stretch peaks at 1063 cm^−1^ and 1130 cm^−1^, respectively, the CH_2_ twist mode at 1296 cm^−1^, and multiple modes associated with CH_2_ bending motion at 1418 cm^−1^, 1441 cm^−1^, and 1464 cm^−1^^32^. On the other hand, interestingly, in the case of the sulfonated fiber at a pressure of 5 bar, the sharp peak such as the C–C symmetric, C–C asymmetric stretch peaks and CH_2_ twist mode disappeared, and a broad G band reflecting an ideal sp^2^ carbon clusters was observed in the 1600 cm^−1^ region. Therefore, based on these Raman results, we assumed that the molecular structure of the polyethylene have completely changed, and as the sulfonation time increases, the G band peak gradually increases, and a weak D band reflecting sp^2^ carbon layers was observed In the case of polyethylene based carbon fiber, remarkable D band peak and G band peak were observed, which can be confirmed similar to the Raman shift of general carbon fiber. In particular, in the case of the D band peak, it can be seen that the carbonization treatment is significantly improved, which means that it is converted to a turbo-stratic structure at a high temperature of 1000 °C^33^.

#### Morphological analysis of the polyethylene-based carbon fibers

Figure S12 shows SEM images of the polyethylene-based carbon fiber prepared from partially drawn LLDPE fiber with a sulfonation pressure of 1 bar at 130 °C for 2.5 h. All fibers had a hollow shape with an empty center part, showing that cracks formed through the axial direction of the carbon fiber. This is because sulfuric acid does not penetrate the fibers and cross-linking reactions by sulfonation proceeded only in the part of the fiber that reacted. Thus, it could not develop into carbon fibers with superior mechanical properties. In addition, in the case of partially drawn sulfonated HDPE fiber subjected to sulfonation under the same sulfonation conditions, the shape of the fiber was not maintained and all decomposed thermally; thus, SEM analysis was impossible.

The polyethylene-based carbon fiber sulfonated under a pressure of 5 bar exhibited different morphological characteristics from polyethylene-based carbon fiber sulfonated at 1 bar.

**Figure 11:**
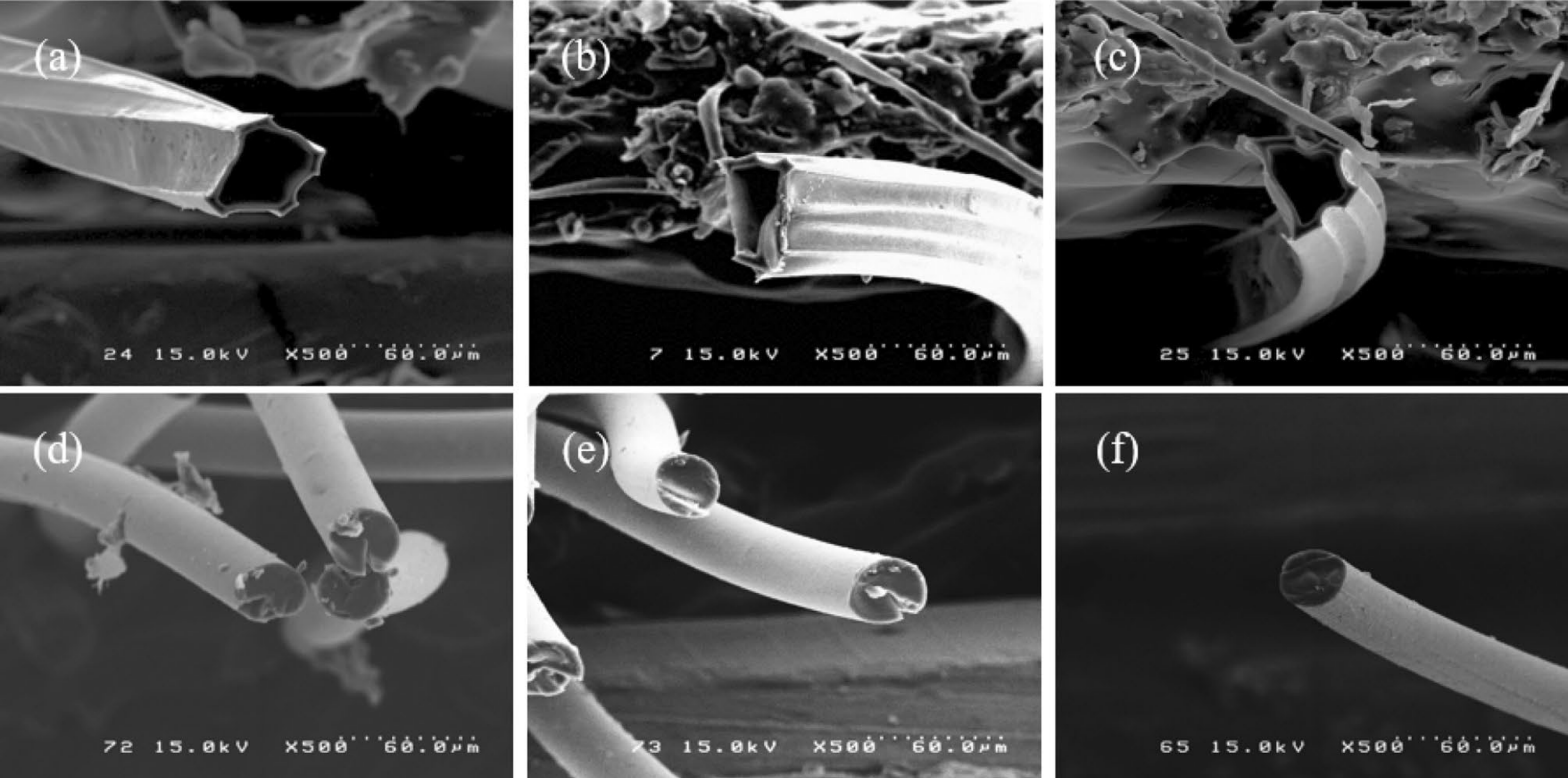
Polyethylene-based carbon fibers prepared from partially drawn HDPE fibers and partially drawn LLDPE fiber with a sulfonation pressure of 5 bar at 130 °C for 3.5 h, 2.5 h (**a**) CH0.6, (**b**) CH7, (**c**) CH18, (**d**) CL1.1, (**e**) CL10, and (**f**) CL20.

Figure 11 shows the polyethylene-based carbon fibers prepared by partially drawn HDPE fibers with sulfonation pressure of 5 bar at 130 °C for 3.5 h and partially drawn LLDPE fiber with sulfonation pressure of 5 bar at 130 °C for 2.5 h. In the case of CH0.6 (Fig. 11a), CH7 (Fig. 11b), and CH18 (Fig. 11c), the carbonized fibers did not maintain a circular cross-section but had a sharp shape, such as a pentagonal structure and a hexagonal structure, as well as a hollow interior. This is because the fiber has an irregular cross-sectional shape and a hollow cross-section due to the poor cross-linked efficiency of the HDPE fiber. Although sulfuric acid permeated into the HDPE fiber under the sulfonation conditions in a high-pressure hydrostatic atmosphere, the cross-linking reaction by SO_3_ did not occur actively because of the molecular structure of the HDPE fiber with only the main chain. In addition, the mechanical properties of the CH0.6, CH7, and CH18 specimens were extremely low and could not be measured.

In the case of the specimen carbonized with partially drawn sulfonated LLDPE fiber, the specimen exhibited a completely different tendency compared to the high-density polyethylene-based carbon fiber. Figure 11d–f present cross-section SEM images of the LLDPE-based carbon fiber. The CL1.1, CL10, and CL20 specimens showed a completely filled fiber cross-section. This means that sulfuric acid diffused and penetrated well into the LLDPE fibers and cross-linked due to the high sulfonation hydrostatic pressure of 5 bar and the molecular structure of the LLDPE fiber, which contained a large number of branches with the polymer main chain. The hollow cross-section was not observed in the CL1.1 and CL10 specimens, but a long crack was formed in the fiber axial direction, which was attributed to residual stress caused by thermal hysteresis in the carbonization process. In contrast, in the CL20 specimen, the cross-section image of the carbon fiber revealed a circular shape, no cracks, and a reasonably clean surface. This indicates that the cross-linking reaction of the partially drawn LLDPE fiber with a melt flow index of 20 was extremely stable during the sulfonation process at 5 bar, 130 °C, and 2.5 h, resulting in an aromatic structure. Furthermore, even in the carbonization process, the fibers could not decompose at high temperatures.

#### Mechanical properties of the polyethylene-based carbon fiber

Figure S13 shows the stress–strain curve of the polyethylene-based carbon fiber (CL20). The tensile strength, tensile modulus, and elongation at break of the carbon fiber, which were confirmed through the stress–strain curve, were 2.03 GPa, 143.63 GPa, and 1.42%, respectively. Carbon fibers exhibited completely elastic properties and high strength, suggesting that the aromatic structure polymer was carbonized at 1000 °C.

#### Cross-link mechanism and carbonization mechanism of the LLDPE fiber and elemental composition.

Figures S14 and S15 show schematic diagrams of the cross-linking mechanism and carbonization mechanism of the poly ethylene-based carbon fibers through polyethylene precursor fiber analysis, mechanical and chemical structure analysis of sulfonated polyethylene fibers, and carbon fiber analysis^18,20,28^. Table S16 lists the compositions of C, H, O, N, and S in the carbon fiber prepared from partially drawn HDPE fibers and fully drawn LLDPE fibers.

#### Proposal of polyethylene based carbon fiber manufactured by conventional sulfonation method and hydrostatic pressure sulfonation method

Figure S16 shows the manufacturing process and schematic diagram of polyethylene based carbon fiber through conventional method and hydrostatic pressure sulfonation method.

## Conclusion

This paper reported the preparation of carbon fibers using HDPE fibers and LLDPE fibers. In the first time, the draw ratio of the polyethylene fibers and the sulfonation mechanism under hydrostatic pressure conditions of 1 bar and 5 bar were investigated.

The influence of the melt flow index of polyethylene on the sulfonation was studied. The LLDPE fibers having a side chain and a low melt flow index were converted successfully to carbon fibers. When the melt flow index value was high, the fluidity of the polymer was improved at a temperature above the melting point of polyethylene, so that precursor fibers with a low diameter could be prepared. It was experimentally confirmed that the lower the diameter of the precursor fiber, the shorter the penetration time of sulfuric acid into the fiber.

Polyethylene fibers, which exhibited thermoplastic properties and plastic behavior, were cross-linked through a sulfonation process. The thermal properties and mechanical properties were changed due to the thermosetting properties and elastic behavior. The transition from aliphatic polymers to aromatic polymers was an important issue. Although sulfonation was performed under a hydrostatic pressure of 5 bar, it was difficult to convert the HDPE and LLDPE fibers to aromatic polymers because of their high crystallinity. Polyethylene with side chains, such as LLDPE rather than HDPE, was more likely to form a benzene ring by crosslinking adjacent aliphatic polymers. It was confirmed that the penetration rate of sulfuric acid was faster under the pressure condition of 5 bar than under the pressure condition of 1 bar. Using a linear low-density polyethylene fiber with a melt flow index of 20 and side chains in the polymer structure that can form a low diameter, sulfonation was performed at 130 °C for 2.5 h under a hydrostatic pressure of 5 bar. This was followed by carbonization under carbonization conditions of 1000 °C, 5 °C/min, and 5 min. The resulting carbon fibers had a tensile strength, tensile modulus, and tensile elongation of 2.03 GPa, 143.63 GPa, and 1.42%, respectively. Therefore, in this paper, polyethylene based carbon fiber exhibiting performance level similar to that of the previously reported literature was prepared, and sulfonation was performed under hydrostatic pressure condition, which has not been attempted so far. In particular, the relationship between fiber orientation and sulfonation efficiency was investigated in detail.

## Supplementary Information

Supplementary Information.
