# Supplementary material for: Study on polyethylene-based carbon fibers obtained by sulfonation under hydrostatic pressure
Source: Sci Rep. 2021 Sep 9;11:18028. doi: 10.1038/s41598-021-97529-4 (PMC8429680; doi:10.1038/s41598-021-97529-4)
Supplement: Supplementary file 1 — Supplementary Information. [file 41598_2021_97529_MOESM1_ESM.docx]

**2.6. Characterization**

**2.6.1. Gel permeation chromatography**

Gel permeation chromatography (GPC, PL-GPC 220 system, USA) analysis was conducted in trichlorobenzene + 0.04% butyrated hydroxytoluene (after drying with 0.1% CaCl_2_) at a flow rate of 1.0mL/min at 160°C. The chromatograph was equipped refractive index detector and a Plgel mixed-b column. Polystyrene standards were employed for molecular weight calibration. The sample injection volume and sample concentration were 200μL and 2mg/mL, respectively, and the molecular weight data were analyzed using Agilent GPC/SEC software. The number average molecular weight (M_n_), weight average molecular weight (M_w_), and polydispersity index (PDI) were calculated relative to polystyrene standards. The PDI was calculated using the following equation.


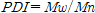
····························································································(1)

**2.6.2. Mechanical properties of the polyethylene precursor fibers**

The mechanical properties of the fully drawn and partially drawn precursor fibers were investigated using a tensile test. The experiments were conducted using the ASTM D-2256 standard procedure. The equipment used to measure the properties was a universal testing machine (UTM, OTT-05, Oriental, Korea). The gauge length of the test specimens was 250mm, and the crosshead speed was 300mm/min; 20 specimens were measured. The tensile strength, tensile modulus, and elongation at break of the polyethylene fiber were calculated using Hooke's law as follows:

$\frac{F}{A}$ = *E*$\varepsilon$ ··································································································· (2)

where *F*, *A*, *E*, and ε are the tensile load, cross-sectional area of the precursor fiber acting perpendicular to the tensile load, initial tensile modulus of the precursor fiber, and elongation at break of the precursor fiber, respectively.

**2.6.3. Fourier transform nuclear magnetic resonance (FT-NMR) analysis**

^1^H NMR spectra were recorded at 600MHz using an FT-NMR spectrometer (VNMR 600MHz, Agilent technologies, USA). Dimethyl sulfoxide (DMSO-d6) was used as a deuterated solvent. The polyethylene precursor fiber and sulfonated polyethylene fiber were dissolved in DMSO-d6 in a vacuum oven at 100°C for three days. Subsequently, 600μL of a solution was placed into a 5mm diameter glass tube using a micro-pipette, and the analysis was performed.

**2.6.4. Scanning electron microscopy (SEM)**

The morphology of the polyethylene precursor fibers, sulfonated fibers, and polyethylene-based carbon fibers was analyzed by SEM (S-4100, Hitachi, LTD., Japan) at × 500 magnification. All specimens were coated with platinum before the SEM observations. SEM was performed to observe the influence of the sulfonation time and pressure conditions on sulfur cross-linking.

**2.6.5. Wide-angle X-ray diffraction (WAXD)**

Wide-angle X-ray diffraction (WAXD, D8 ADVANCE, Bruker, USA) was used to determine the orientation of the polyethylene precursor fiber and sulfonated polyethylene fiber using Cu Kα radiation (λ = 0.154nm) at 40kV and 40mA.

**2.6.6. Differential scanning calorimetry (DSC) analysis**

The melting enthalpy and melting temperature were measured by DSC (Diamond DSC, Perkin Elmer, Inc. USA). The specimens (2∼3 mg) were encapsulated in aluminum pans and treated at a heating rate of 10°C/min. The atmosphere was nitrogen with a flow rate of 45 mL/min. The influence of cross-linking by sulfuric acid on the fully drawn and partially drawn polyethylene fibers was examined. The analytical temperature range was set between 40°C and 140°C.

**2.6.7. Mechanical properties of the sulfonated fibers and polyethylene-based carbon fibers**

The mechanical properties of the sulfonated polyethylene fibers and polyethylene-based carbon fibers were investigated using a tensile test. The experiments were conducted using the ASTM D-3379 standard procedure. The equipment used to measure the properties was a universal testing machine (UTM, OTT-05, Oriental, Korea). The gauge length of the test specimens was 25 mm, and the crosshead speed was 0.1mm/min; 20 specimens were measured. The tensile strength, tensile modulus, and elongation at break value of sulfonated polyethylene fiber were calculated using Hooke's law (equation 2).

**2.6.8. Fourier transform infrared (FTIR) analysis**

FTIR spectroscopy was performed to observe the changes in the chemical structure of the fully drawn and partially drawn fibers, such as HDPE and LLDPE fibers, according to the sulfonation time. FT-IR (Perkin Elmer Co. model spectrum 100, USA) spectroscopy was performed from 500 to 3000 cm^-1^. The LLDPE and HDPE fibers were sulfonated for 2.5 and 3.5 hours, respectively, and analyzed. The chemical structure was compared with pristine polyethylene fibers without the sulfonation process.

**2.6.9. Elemental analysis of the precursor fibers, sulfonated polyethylene fibers, and polyethylene-based carbon fibers**

Elemental analysis was performed using an EA 2000 elemental analyzer (EA 2000, Thermo Fisher Scientific Co., USA). The presence of C, H, O, N, and S was analyzed in the polyethylene-based carbon fiber using a tungstic anhydride catalyst at 1000°C. The O concentration was analyzed using a nickel-plated carbon catalyst at 1060°C. BBOT, L-cystine, and sulfanilamide were used as standard materials.

**2.6.10. Raman spectroscopy analysis**

Raman measurements were carried out on a Jobin Yvon Horiba LabRAM HR Evolution spectrometer (Horiba scientific Co., Japan) using a 1800grooves/mm grating and 300$\mu$m confocal pinhole setting with a 532nm laser. The laser was focused through 100X optical lens and exposure time was set as 2s.

**Table S1.** Previously reported polyethylene-based carbon fiber.

| **Previous report** | **Materials** | **Precursor fiber diameter** | **Tensile strength**  **and modulus of**  **the precursor fiber** | **Sulfonation condition** | **Carbon fiber diameter** | **Tensile strength**  **and modulus of**  **the carbonized fiber** |
| --- | --- | --- | --- | --- | --- | --- |
| A.Postema, et al  J. Mater. Sci.  1990;25(10):4216-4222 | LLDPE  Tm :127.6 °C | 85 $\mu$m | 0.25 GPa  18 GPa | HSO_3_Cl 98%  RT  0 ~ 40 hour  1bar | 40 $\mu$m | 1.15 GPa  60 GPa  3.0 % |
| D. Zhang  J.Thermo. Compos. Mater.  1993;6(1):38-48 | UHMWPE  Tm : 150 °C | Unknown | Unknown | H_2_SO_4_ 95%  130 ~ 200 °C  5 ~ 120 min  1bar | Unknown | 2.1 GPa  210 GPa  1.0 % |
| D. Zhang, G. Bhat  Mater. Manuf. Process.  1994;9(2):221-235 | UHMWPE | 33 $\mu$m | 0.33 GPa, 0.26 Gpa  170 Gpa, 120 GPa  2.5%, 3.5% | H_2_SO_4_ 95%  130 ~ 200 °C  15 ~ 120 min  1bar | Unknown | 2.1 GPa  210 GPa  1.0 % |
| D. Zhang, Q. Sun  J. Appl. Polym. Sci.  1996;62(2):367-  373 | Unknown | Unknown | Unknown | H_2_SO_4_ 95%  130 ~ 180 °C  5 ~ 90 min  1bar | Unknown | 2.0 GPa  200 GPa  1.0 % |
| M. Hunt, T. Saito et al.  Advanced Materials  2012;24(18):2386-2389 | LLDPE  Unknown | 16 ~ 19 $\mu$m | 0.10 ~ 0.17 GPa  0.14 ~ 1.03 GPa  100 ~ 190 % | H_2_SO_4_ (SO_3_)x 120%  70 °C  Unknown  1bar | 15 $\mu$m | 0.63 - 1.1 GPa  27.6 - 103.4 GPa  1.1 - 1.6 % |
| J. Kim, J. Lee et al.  Carbon  2015;94:524-530 | LLDPE  Tm : 122 °C  MI : 1.1g/10min | 27$\mu$m  (Solution spinning) | 0.14 GPa  0.1 GPa | H_2_SO_4_ 95%  130 ~ 160 °C  60 ~ 240 min  1bar | 15 $\mu$m | 1.65 GPa  110 GPa |
| G. Wortberg, et al.  Fibers  2015;3(3):373-379 | HDPE  Tm : 190 °C  MI : 20 g/10min | 16.4 ~ 40.7 $\mu$m | Unknown | Unknown | Unknown | Unknown |
| A. De Palmenaer et al.  Chem. Eng. Trans.  2015;43:1699-1704 | HDPE  Tm : 190 °C  MI : 28 g/10min | 15 ~ 17 $\mu$m | Unknown | H_2_SO_4_ 95%  90 ~ 180 °C  0 ~ 135 min  1bar | Unknown | Unknown |
| M. Behr, B. Landes et al.  Carbon  2016;107:525-535 | Unknown | 10 $\mu$m | Unknown | H_2_SO_4_(SO_3_)x, H_2_SO_4_ 96%  50 ~ 140 °C  30 ~ 180 min  1bar | 6 ~ 19 $\mu$m | 2.1 GPa  390 GPa |
| B. Barton, et al.  Small  2017;13(36):1-7 | LLDPE  Tm : 131 °C  MI : 30 g/10min | 10 $\mu$m | Unknown | H_2_SO_4_ (SO_3_)x, H_2_SO_4_  Aqueous boric acid solutions 50 °C ~ 140°C 120 min  1bar | 6 $\mu$m | 2.4 GPa  200 GPa  1.7 % |
| D. Choi, S. Yoo, S. Lee  Carbon  2019;146:9-16 | LLDPE  Tm : 129 °C  MI : 30 g/10min | 14 $\mu$m | 0.1 GPa  0.7 GPa | H_2_SO_4_ 98%  95 °C  90 min  1bar | 12 $\mu$m | 1.3 GPa  89.8 GPa  1.5 % |
| ***This study*** | HDPE, LLDPE  Tm : 106 ~ 134  MI : 0.6 ~ 20 | 18 ~ 60 $\mu$m | 0.09 ~ 0.64 GPa  0.62 ~ 6.40 GPa | H_2_SO_4_ 98%  80°C for 4h  130°C for 2.5h  1 ~ 5bar | 15 $\mu$m | 2.03 Gpa  143.6 Gpa  1.4 % |

| **Materials** | **HDPE** | **HDPE** | **HDPE** | **LLDPE** | **LLDPE** | **LLDPE** |
| --- | --- | --- | --- | --- | --- | --- |
| **Manufacturer** | LG  Chem | LOTTE Chem | LOTTE Chem | LOTTE Chem | LOTTE Chem | LOTTE Chem |
| **Product number** | SP380 | 2210J | 2600F | UN324 | UL614 | UL814 |
| **Melting index (g/10min)** | 0.6 | 7 | 18 | 1.1 | 10 | 20 |
| **Density (g/cm^3^)** | 0.952 | 0.959 | 0.959 | 0.923 | 0.924 | 0.924 |
| **Tensile strength (MPa)** | 10 | 22.6 | 20 | 39.2 | 10.3 | 10.3 |
| **Elongation at break (%)** | 1000 | 500 | 300 | 750 | 500 | 500 |
| **Melting temperature (**$\boldsymbol{^{\circ}}$**C)** | 134 | 132 | 130 | 125 | 123 | 106 |
| **Softening point (**$\boldsymbol{^{\circ}}$**C)** | 127 | 122 | 120 | 94 | 93 | 92 |

**Table S2.** Specifications of the HDPE pellets and LLDPE pellets.

**Table S3.** Spinning conditions of the polyethylene precursor fibers and drawing conditions of the polyethylene precursor fibers.

| **Spinning conditions of the precursor fiber** | | | | | | |
| --- | --- | --- | --- | --- | --- | --- |
| **Materials** | **HDPE0.6** | **HDPE7** | **HDPE10** | **LLDPE1.1** | **LLDPE10** | **LLDPE20** |
| **Spinning distance (mm)** | 530 | | | | | |
| **Spinning speed (m/min)** | 0.1 | | | | | |
| **Spinning temperature (**$\boldsymbol{^{\circ}}$**C)** | 160 | | | 150 | | |
| **1^st^ roller speed (m/min)** | 10 | 10 | 10 | 10 | 10 | 10 |
| **2^nd^ roller speed (m/min)** | 20 | 20 | 20 | 20 | 20 | 20 |
| **Take-up speed (m/min)** | 33 | 35 | 40 | 33 | 35 | 40 |
| **Spinning nozzle diameter (mm)** | 2 | 2 | 2 | 2 | 1 | 1 |
| **Fiber diameter (**$\boldsymbol{\mu}$**m)** | 60 | 40 | 30 | 34 | 27 | 22 |
| **Drawing conditions of the precursor fiber** | | | | | | |
| **Materials** | **HDPE0.6** | **HDPE7** | **HDPE10** | **LLDPE1.1** | **LLDPE10** | **LLDPE20** |
| **Drawing temperature (**$\boldsymbol{^{\circ}}$**C)** | 80 | | | | | |
| **Heat treatment time at 80**$\boldsymbol{^{\circ}}$**C before and after drawing (min)** | 10 | | | | | |
| **Drawing speed (m/min)** | 0.021 | | | | | |
| **Draw ratio** | 4.1 | | 5.2 | 4.4 | 5.2 | |
| **Fiber diameter (**$\boldsymbol{\mu}$**m)** | 40 | 26 | 20 | 28 | 20 | 18 |

**Table S4.** Specimen code of the polyethylene precursor fibers, sulfonated polyethylene fibers and carbonized polyethylene fibers.

| **Specimen code of the polyethylene precursor fibers** | | | | | | | | | | | | | | |
| --- | --- | --- | --- | --- | --- | --- | --- | --- | --- | --- | --- | --- | --- | --- |
| **Specimen code** | **PH0.6** | **PH7** | | **PH18** | **FH0.6** | **FH7** | **FH18** | **PL1.1** | **PL10** | | **PL20** | **FL1.1** | **FL10** | **FL20** |
| **Materials** | HDPE fiber | | | | | | | LLDPE fiber | | | | | | |
| **MI value** | 0.6 | 7 | | 18 | 0.6 | 7 | 18 | 1.1 | 10 | | 20 | 1.1 | 10 | 20 |
| **Degree of drawing** | Partially drawn fiber | | | | Fully drawn fiber | | | Partially drawn fiber | | | | Fully drawn fiber | | |
| **Specimen code of the sulfonated polyethylene fibers** | | | | | | | | | | | | | | |
| **Specimen code** | **PSH0.6** | **PSH7** | | **PSH18** | **FSH0.6** | **FSH7** | **FSH18** | **PSL1.1** | **PSL10** | | **PSL20** | **FSL1.1** | **FSL10** | **FSL20** |
| **Materials** | Sulfonated HDPE fiber | | | | | | | Sulfonated LLDPE fiber | | | | | | |
| **MI value** | 0.6 | 7 | | 18 | 0.6 | 7 | 18 | 1.1 | 10 | | 20 | 1.1 | 10 | 20 |
| **Degree of drawing** | Partially drawn fiber | | | | Fully drawn fiber | | | Partially drawn fiber | | | | Fully drawn fiber | | |
| **Specimen code of the carbonized polyethylene fibers** | | | | | | | | | | | | | | |
| **Specimen code** | **CH0.6** | | | **CH7** | | **CH18** | | **CL1.1** | | **CL10** | | | **CL20** | |
| **Materials** | HDPE based carbon fiber | | | | | | | LLDPE based carbon fiber | | | | | | |
| **Melt flow index** | 0.6 | | 7 | | | 18 | | 1.1 | | 10 | | | 20 | |
| **Carbonized conditions** | 1000$^{\circ}$C, 5$^{\circ}$C/min, 5min | | | | | | | | | | | | | |

**Table S5.** Tensile strength, initial tensile modulus, and elongation at break values of fully drawn polyethylene precursor fiber with different draw ratio.

|  | **Tensile strength**  **(GPa)** | **Initial modulus**  **(GPa)** | **Elongation at break**  **(%)** |
| --- | --- | --- | --- |
| **FH 0.6** | 0.64 | 6.40 | 10.21 |
| **FH 7** | 0.38 | 5.65 | 13.79 |
| **FH 18** | 0.33 | 4.40 | 15.77 |
| **FL 1.1** | 0.39 | 6.18 | 9.66 |
| **FL 10** | 0.16 | 3.92 | 12.60 |
| **FL 20** | 0.09 | 3.73 | 13.34 |

**Table S6.** Tensile strength, initial tensile modulus, and elongation at break values of partially drawn polyethylene precursor fiber with different draw ratio.

|  | **Tensile strength**  **(GPa)** | **Initial modulus**  **(GPa)** | **Elongation at break**  **(%)** |
| --- | --- | --- | --- |
| **PH 0.6** | 0.21 | 1.28 | 102.10 |
| **PH 7** | 0.13 | 1.13 | 129.63 |
| **PH 18** | 0.11 | 0.88 | 134.05 |
| **PH 1.1** | 0.13 | 1.24 | 96.60 |
| **PH 10** | 0.12 | 0.63 | 134.34 |
| **PH 20** | 0.10 | 0.62 | 136.51 |

**Table S7.** Diameter, length, and weight values of the HDPE and LLDPE fibers according to the sulfonation time under 1 bar pressure.

|  | | **Partially drawn fiber** | | | | | **Fully drawn fiber** | |
| --- | --- | --- | --- | --- | --- | --- | --- | --- |
|  |  | 0h | 2h | 2.5h | 3.5h | 5h | 2.5h | 3.5h |
| **HDPE 0.6** | Diameter ($\mu$m) | 60 | 70 | - | 80 | 98 | - | 71 |
|  | Length (mm) | 500 | 431 | - | 354 | 297 | - | 332 |
| **HDPE 7** | Diameter ($\mu$m) | 40 | 47 | - | 52 | 60 | - | 47 |
|  | Length (mm) | 500 | 443 | - | 374 | 314 | - | 351 |
| **HDPE 18** | Diameter ($\mu$m) | 30 | 37 | - | 40 | 45 | - | 31 |
|  | Length (mm) | 500 | 451 | - | 398 | 334 | - | 381 |
| **LLDPE 1.1** | Diameter ($\mu$m) | 34 | 37 | 41 | - | 44 | 39 | - |
|  | Length (mm) | 500 | 457 | 419 | - | 364 | 397 | - |
| **LLDPE 10** | Diameter ($\mu$m) | 27 | 29 | 33 | - | 35 | 31 | - |
|  | Length (mm) | 500 | 464 | 421 | - | 371 | 401 | - |
| **LLDPE 20** | Diameter ($\mu$m) | 22 | 24 | 25 | - | 28 | 24 | - |
|  | Length (mm) | 500 | 478 | 432 | - | 394 | 413 | - |

**Table S8.** Diameter, length, and weight values of the HDPE and LLDPE fibers according to the sulfonation time under 5 bar pressure.

|  | | **Partially drawn fiber** | | | | | **Fully drawn fiber** | |
| --- | --- | --- | --- | --- | --- | --- | --- | --- |
|  |  | 0h | 2h | 2.5h | 3.5h | 5h | 2.5h | 3.5h |
| **HDPE 0.6** | Diameter ($\mu$m) | 60 | 63 | - | 67 | 70 | - | 59 |
|  | Length (mm) | 500 | 472 | - | 451 | 432 | - | 430 |
| **HDPE 7** | Diameter ($\mu$m) | 40 | 43 | - | 45 | 50 | - | 40 |
|  | Length (mm) | 500 | 478 | - | 462 | 442 | - | 447 |
| **HDPE 18** | Diameter ($\mu$m) | 30 | 32 | - | 33 | 35 | - | 28 |
|  | Length (mm) | 500 | 484 | - | 472 | 457 | - | 467 |
| **LLDPE 1.1** | Diameter ($\mu$m) | 34 | 35 | 36 | - | 37 | 34 | - |
|  | Length (mm) | 500 | 494 | 487 | - | 481 | 473 | - |
| **LLDPE 10** | Diameter ($\mu$m) | 27 | 28 | 29 | - | 30 | 27 | - |
|  | Length (mm) | 500 | 496 | 490 | - | 487 | 484 | - |
| **LLDPE 20** | Diameter ($\mu$m) | 22 | 23 | 24 | - | 25 | 24 | - |
|  | Length (mm) | 500 | 496 | 492 | - | 490 | 487 | - |

**Table S9.** Melting temperature and endothermic enthalpy values of the fully-drawn sulfonated high density polyethylene fibers with melt flow index of 0.6,7 and 18 according to the various sulfonation time under pressure of 5bar and temperature of 130°C

| **Specimen code** | **Melting temperature (℃)** | **Endothermic enthalpy (J/g)** |
| --- | --- | --- |
| **FSH18 5h** | 128.48 | 0.28 |
| **FSH18 3.5h** | 129.41 | 7.47 |
| **FSH18 2h** | 130.29 | 22.60 |
| **FH18** | 132.57 | 47.40 |
| **FSH7 5h** | 130.21 | 3.05 |
| **FSH7 3.5h** | 131.21 | 34.88 |
| **FSH7 2h** | 132.43 | 42.22 |
| **FH7** | 135.36 | 139.36 |
| **FSH0.6 5h** | 135.21 | 7.37 |
| **FSH0.6 3.5h** | 132.21 | 40.40 |
| **FSH0.6 2h** | 136.36 | 102.82 |
| **FH0.6** | 137.57 | 174.36 |

**Table S10.** Melting temperature and endothermic enthalpy values of the partially-drawn sulfonated high density polyethylene fibers with melt flow index of 0.6,7 and 18 according to the various sulfonation time under pressure of 5bar and temperature of 130°C

| **Specimen code** | **Melting temperature (℃)** | **Endothermic enthalpy (J/g)** |
| --- | --- | --- |
| **PSH18 5h** | - | - |
| **PSH 18 3.5h** | - | - |
| **PSH18 2h** | 130.19 | 2.788 |
| **PH18** | 130.5 | 39.62 |
| **PSH7 5h** | - | - |
| **PSH7 3.5h** | - | - |
| **PSH7 2h** | 131.43 | 15.55 |
| **PH7** | 132.50 | 45.18 |
| **PSH0.6 5h** | - | - |
| **PSH0.6 3.5h** | 132.79 | 7.79 |
| **PSH0.6 2h** | 134.10 | 57.12 |
| **PH0.6** | 135.21 | 119.96 |

**Table S11.** Melting temperature and endothermic enthalpy values of the fully-drawn sulfonated linear low density polyethylene fibers with melt flow index of 1.1,10 and 20 according to the various sulfonation time under pressure of 5bar and temperature of 130°C.

| **Specimen code** | **Melting temperature (℃)** | **Endothermic enthalpy (J/g)** |
| --- | --- | --- |
| **FSL20 5h** | - | - |
| **FSL20 2.5h** | - | - |
| **FSL20 2h** | 120.03 | 8.33 |
| **FL20** | 122.67 | 92.6 |
| **FSL10 5h** | - | - |
| **FSL10 2.5h** | 121.11 | 34.5 |
| **FSL10 2h** | 122.11 | 55.08 |
| **FL10** | 123.48 | 68.54 |
| **FSL1.1 5h** | 120.5 | 20.4 |
| **FSL1.1 2.5h** | 121.08 | 55.14 |
| **FSL1.1 2h** | 122.11 | 103.68 |
| **FL1.1** | 125.54 | 190.52 |

**Table S12.** Melting temperature and endothermic enthalpy values of the partially-drawn sulfonated linear low density polyethylene fibers with melt flow index of 1.1,10 and 20 according to the various sulfonation time under pressure of 5bar and temperature of 130°C.

| **Specimen code** | **Melting temperature (℃)** | **Endothermic enthalpy (J/g)** |
| --- | --- | --- |
| **PSL20 5h** | - | - |
| **PSL20 2.5h** | - | - |
| **PSL20 2h** | 120.01 | 2.16 |
| **PL20** | 121.09 | 34.08 |
| **PSL10 5h** | - | - |
| **PSL10 2.5h** | 120.74 | 6.00 |
| **PSL10 2h** | 121.85 | 7.04 |
| **PL10** | 122.43 | 40.42 |
| **PSL1.1 5h** | - | - |
| **PSL1.1 2.5h** | 120.08 | 1.34 |
| **PSL1.1 2h** | 121.21 | 41.74 |
| **PL1.1** | 123.50 | 140.42 |

**Table S13. ^1^**H NMR spectrum chemical shift and integration values of the partially drawn HDPE fiber and partially drawn LLDPE fiber.

|  | Chemical Shift (ppm) | Integration |
| --- | --- | --- |
| HDPE 0.6 | 1.239 | 414.75 |
|  | 1.355 | 100 |
| HDPE 7 | 1.240 | 873.23 |
|  | 1.355 | 100 |
| HDPE 18 | 1.239 | 617.13 |
|  | 1.399 | 100 |
| LLDPE 1.1 | 1.255 | 122.78 |
|  | 1.342 | 181.63 |
|  | 1.410 | 100 |
| LLDPE 10 | 1.251 | 108.68 |
|  | 1.299 | 139.78 |
|  | 1.338 | 188.26 |
|  | 1.342 |  |
|  | 1.412 | 100 |
| LLDPE 20 | 1.241 | 484.19 |
|  | 1.254 |  |
|  | 1.263 |  |
|  | 1.303 | 117.17 |
|  | 1.340 | 100 |

**Table S14. ^1^**H NMR spectrum chemical shift and integration values of the partially drawn sulfonated HDPE and partially drawn sulfonated LLDPE fiber under a sulfonation pressure of 1bar.

|  | Chemical Shift (ppm) | Integration |
| --- | --- | --- |
| SHDPE0.6 (1 bar) | 1.235 | 77.47 |
|  | 1.298 | 100 |
|  | 8.196 | 2.71 |
| SHDPE7 (1 bar) | 1.235 | 67.97 |
|  | 1.297 | 100 |
|  | 8.195 | 10.54 |
| SHDPE18 (1 bar) | 1.297 | 100 |
|  | 8.196 | 36.22 |
| SLLDPE1.1 (1 bar) | 1.243 | 827.89 |
|  | 1.358 | 100 |
|  | 6.0-8.2 | 61.40 |
| SLLDPE10 (1 bar) | 1.231 | 290.04 |
|  | 1.242 |  |
|  | 1.250 |  |
|  | 1.255 | 110.14 |
|  | 1.265 |  |
|  | 1.304 | 100 |
|  | 6.6-8.4 | 170.28 |
| SLLDPE20 (1 bar) | 1.232 | 2270.58 |
|  | 1.242 |  |
|  | 1.256 | 406.51 |
|  | 1.265 |  |
|  | 1.290 | 283.77 |
|  | 1.304 |  |
|  | 1.342 | 100 |
|  | 6.6-8.2 | 236.41 |

**Table S15. ^1^**H NMR spectrum chemical shift and integration values of the partially drawn sulfonated HDPE fiber and partially drawn sulfonated LLDPE fiber under a sulfonation pressure of 5 bar.

|  | Chemical Shift (ppm) | Integration |
| --- | --- | --- |
| SHDPE0.6 (5 bar) | 1.291 | 100 |
|  | 8.194 | 23.81 |
| SHDPE7 (5 bar) | 1.282 | 100 |
|  | 8.192 | 59.18 |
| SHDPE18 (5 bar) | 1.275 | 100 |
|  | 8.196 | 90.46 |
| SLLDPE1.1 (5 bar) | 1.275 | 100 |
|  | 8.196 | 90.46 |
| SLLDPE10 (5 bar) | 1.240 | 100 |
|  | 8.172 | 384.85 |
| SLLDPE20 (5 bar) | 1.338 | 137.18 |
|  | 1.343 |  |
|  | 1.396 | 100 |
|  | 1.407 |  |
|  | 8.195 | 3414.61 |

**Table S16.** Compositions of C, H, O, N, and S of carbon fibers prepared from partially drawn HDPE fiber and fully drawn LLDPE fiber.

| **Specimen code** | **Element** | **Composition (%)** |
| --- | --- | --- |
| **CH0.6** | N (Nitrogen) | - |
|  | C (Carbon) | 72.95 |
|  | H (Hydrogen) | 10.92 |
|  | S (Sulfur) | 3.50 |
|  | O (Oxygen) | - |
| **CH7** | N (Nitrogen) | - |
|  | C (Carbon) | 85.15 |
|  | H (Hydrogen) | 1.56 |
|  | S (Sulfur) | 2.75 |
|  | O (Oxygen) | - |
| **CH18** | N (Nitrogen) | - |
|  | C (Carbon) | 86.70 |
|  | H (Hydrogen) | 1.57 |
|  | S (Sulfur) | 2.59 |
|  | O (Oxygen) | - |
| **CL1.1** | N (Nitrogen) | - |
|  | C (Carbon) | 87.93 |
|  | H (Hydrogen) | 1.59 |
|  | S (Sulfur) | 2.60 |
|  | O (Oxygen) | - |
| **CL10** | N (Nitrogen) | - |
|  | C (Carbon) | 90.26 |
|  | H (Hydrogen) | 1.56 |
|  | S (Sulfur) | 2.05 |
|  | O (Oxygen) | - |
| **CL20** | N (Nitrogen) | - |
|  | C (Carbon) | 90.43 |
|  | H (Hydrogen) | 1.59 |
|  | S (Sulfur) | 1.88 |
|  | O (Oxygen) | - |


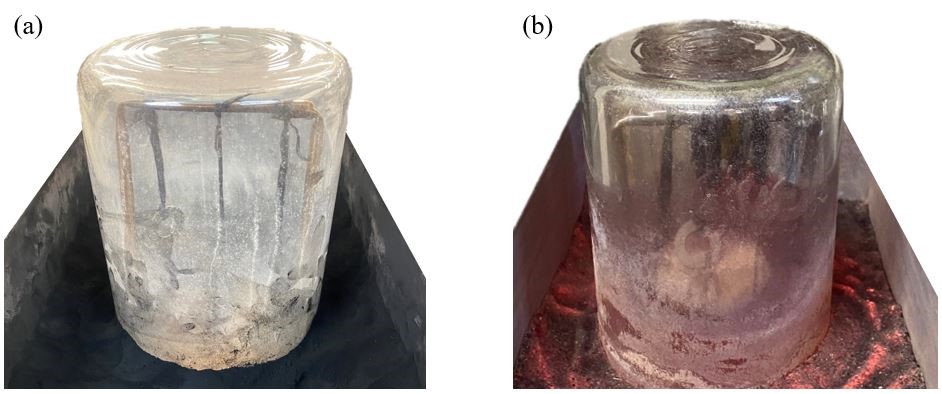


**Figure S1.** Photographs of the carbonization process (a) Before carbonization, (b) After carbonization.


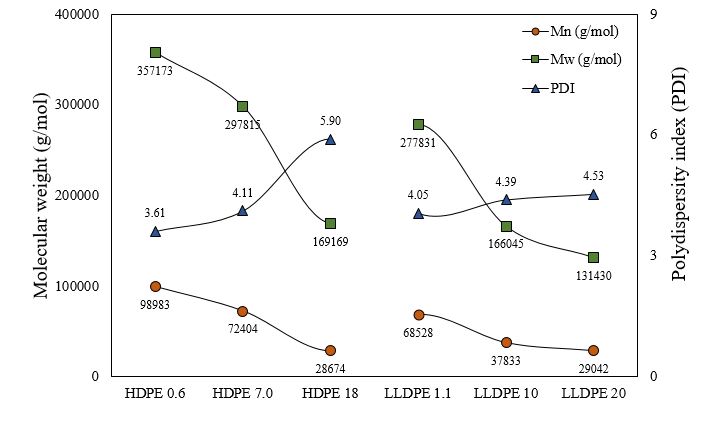
 **Figure S2.** Number average molecular weight, weight average molecular weight and polydispersity index of the polyethylene pellets.


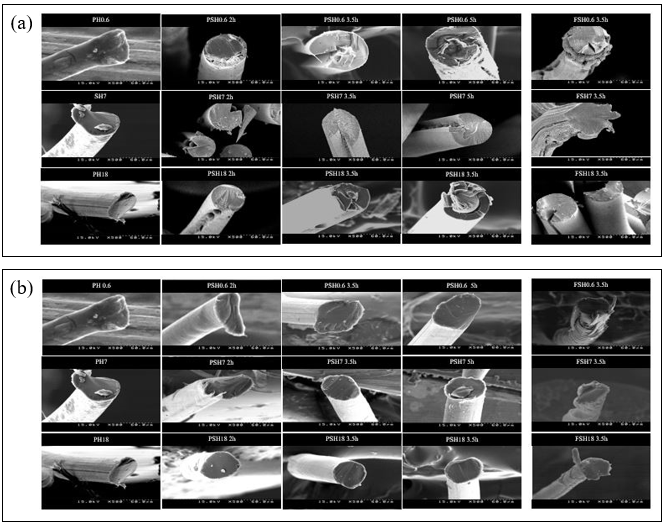


**Figure S3.** SEM images of the cross-sectional surface of the HDPE fibers with melt flow index of 0.6, 7, and 18 according to the sulfonation time under a pressure of 1 bar, 5bar and temperature of 130°C (a) Sulfonated fiber under 1bar pressure, (b) Sulfonated fiber under 5bar pressure.

Figure S3 shows the cross-section SEM images of the sulfonated HDPE fibers. Figure S3 (a) shows cross-section SEM images of the HDPE fibers with a melt flow index of 0.6, 7, and 18 according to the various sulfonation time under a pressure of 1 bar and a temperature of 130°C. After the sulfonation treatment, the diameters of all HDPE fibers increased gradually with increasing sulfonation time. This increase in fiber diameter was attributed to the shrinkage of the longitudinal direction of the HDPE fiber due to prolonged exposure to sulfuric acid during the sulfonation process at 130°C. The HDPE fibers formed a sheath-core structure or cracks formed in the fiber axial direction under all sulfonation time conditions. The failure mechanism changed gradually from plastic deformation to elastic deformation as HDPE fibers cross-link through the sulfonation process. In this process, when shrinkage occurs in the fiber axial direction, residual stress remains in the fiber. Owing to this stress, the fiber with elastic properties does not maintain its original shape and expands, thereby forming cracks. The formation of the core structure of the fiber was attributed to the molecular structure of the HDPE without branches, and the sulfuric acid cannot penetrate the fibers with large diameters during the sulfonation process. HDPE with a molecular structure without side chains (branches) means that it takes a long time to sulfonate the fibers because of the poor cross-linking efficiency. In the case of the fully drawn HDPE fibers, phenomena, such as the core structure of cracks occurring in the fiber axial direction, were more prominent than that of partially drawn HDPE fiber. This is because sulfuric acid cannot penetrate the fiber because of the increase in the crystalline region of the highly oriented fiber. On the other hand, the same type of HDPE fiber specimens, such as PH0.6, PH7, and PH18, exhibited completely different tendencies when sulfonation was performed under a relatively high pressure of 5 bar (Figure S3(b)). After the sulfonation treatment, the diameters of all HDPE fibers increased gradually with increasing sulfonation time, but the degree of the increase in diameter was relatively low compared to the fibers sulfonated under a 1 bar pressure. This is because the hydrostatic pressure prevents the fiber shrinkage phenomenon. In addition, although the partially drawn polyethylene fiber was treated for a long time, such as 5 h, a clean surface without a -core structure was formed, and no cracks were observed in the fiber axial direction. This is because of the faster diffusion rate of the sulfuric acid into the HDPE fiber under a 5 bar hydrostatic pressure than at 1 bar so that residual stress is decreased. In the case of fully drawn HDPE fibers, phenomena, such as the core structure of cracks occurring in the fiber axial direction, were more prominent than the partially drawn HDPE fibers. This is because sulfuric acid could not penetrate the fiber because of the increase in the crystalline region of the highly oriented fiber. In the case of fully drawn HDPE fiber, phenomena, such as the core structure of cracks occurring in the fiber axial direction, still exist. Overall, the degree of crystallinity of the fibers due to the stretching of the HDPE fibers significantly affected the sulfonation process. In contrast to the HDPE fiber specimens, the LLDPE fiber specimens generally did not show cores because the diameter of the fiber was relatively small. Hence, sulfuric acid can penetrate the LLDPE fibers easily. LLDPE with a molecular structure with side chains (branches) allows sulfonation of the fibers within a short time due to good cross-linking efficiency. In the case of fully drawn LLDPE fibers, cracks occurring through the fiber axial direction were more prominent than those of partially drawn LLDPE fibers. Fiber expansion occurred because of the increased residual stress during the change in properties of the LLDPE fiber from plastic behavior to elastic behavior during the sulfonation process.


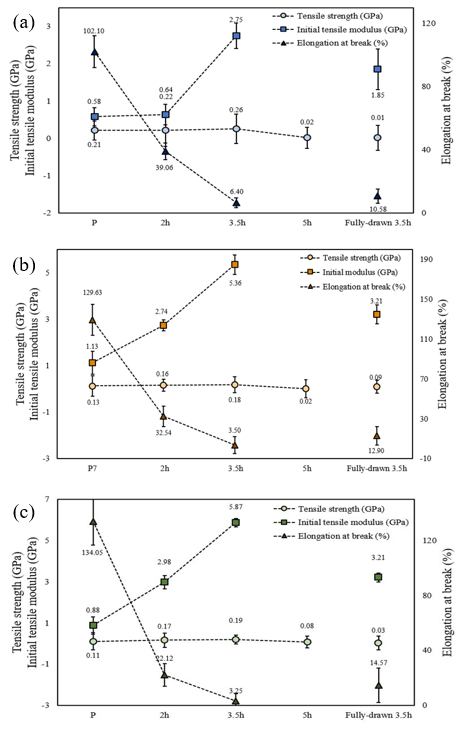


**Figure S4.** Tensile strength, initial tensile modulus, and elongation at break values of the sulfonated HDPE fiber with various sulfonation time under a pressure of 5 bar and temperature of 130°C (a) Sulfonated HDPE fiber with 0.6 melt flow index, (b) Sulfonated HDPE fiber with 7 melt flow index, (c) Sulfonated HDPE fiber with 18 melt flow index.


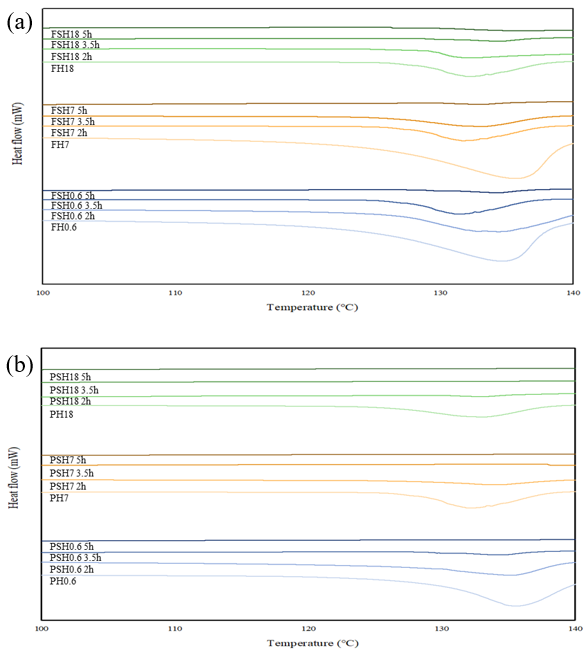


**Figure S5.** DSC curves of the HDPE fibers with melt flow index of 0.6, 7, and 18 according to the sulfonation time under a pressure of 5 bar and temperature of 130°C (a) Fully drawn sulfonated HDPE fiber, (b) Partially drawn sulfonated HDPE fibers.


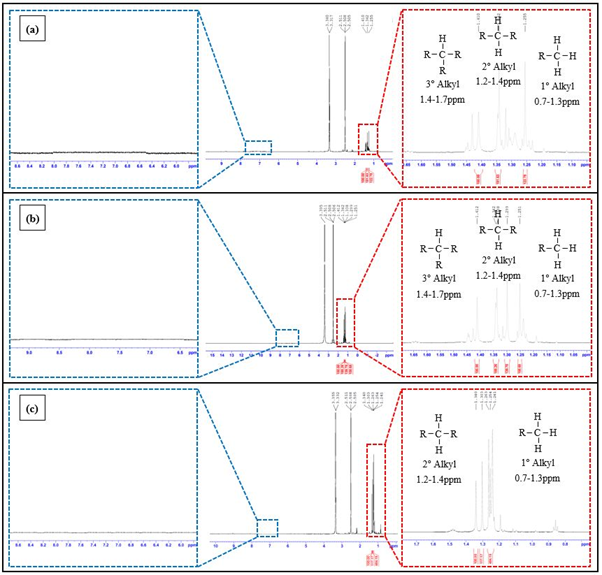


**Figure S6.** ^1^H NMR spectrum and hydrogen peak assignments of partially drawn LLDPE precursor fibers (a) PL1.1, (b) PL10, (c) PL20.


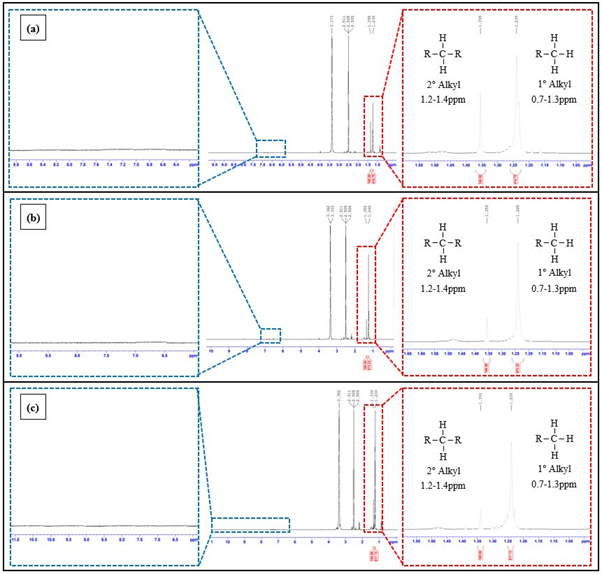


**Figure S7.** ^1^H NMR spectrum and hydrogen peak assignments of partially drawn HDPE precursor fibers (a) PH0.6, (b) PH7, (c) PH18.


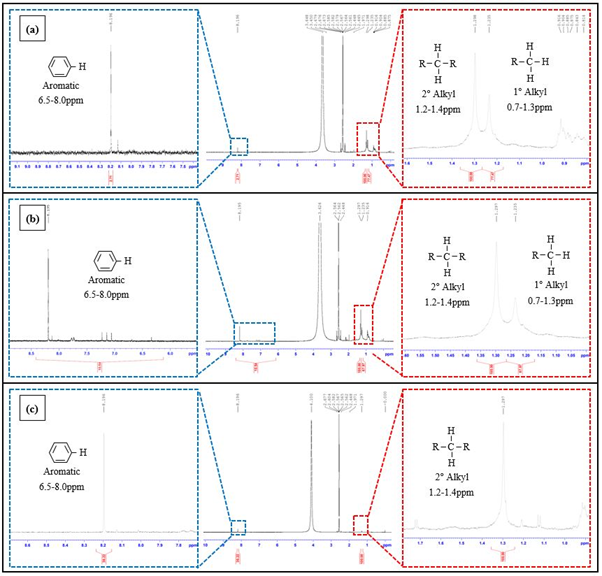


**Figure S8.** ^1^H NMR spectrum and hydrogen peak assignments of sulfonated partially drawn HDPE fibers under sulfonation conditions of 1 bar for 3.5h (a) PSH0.6, (b) PSH7, (c) PSH18.


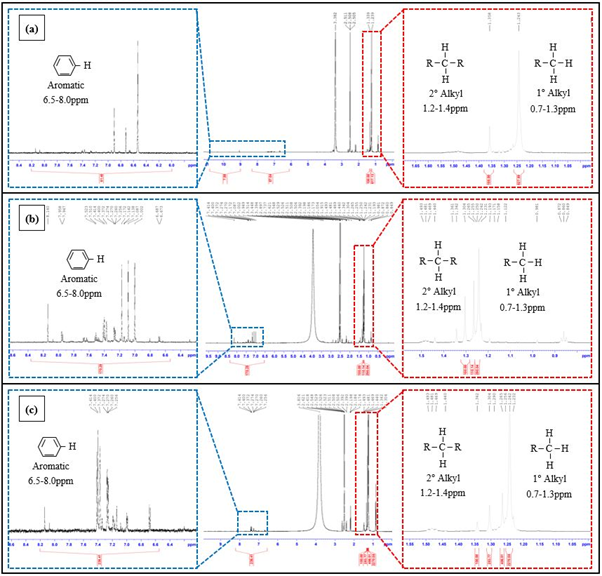


**Figure S9.** ^1^H NMR spectrum and hydrogen peak assignments of sulfonated partially drawn LLDPE fibers under sulfonation conditions of 1 bar for 2.5h (a) PSL1.1, (b) PSL10, (c) PSL20.


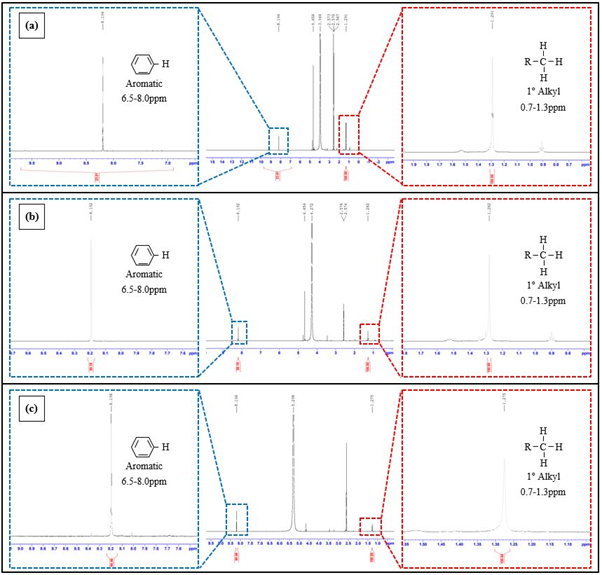


**Figure S10.** ^1^H NMR spectrum and hydrogen peak assignments of sulfonated partially drawn HDPE fibers under sulfonation conditions of 5 bar for 3.5h (a) PSH0.6, (b) PSH7, (c) PSH18.


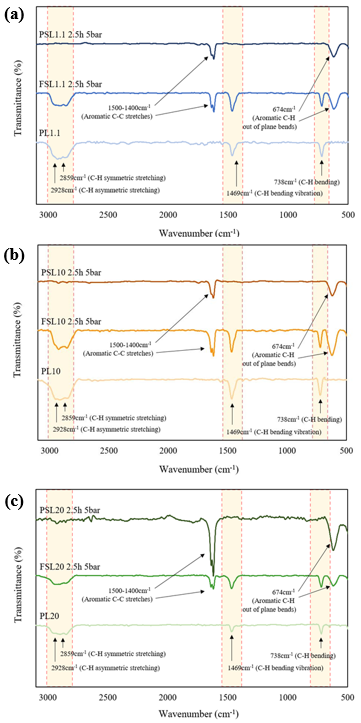


**Figure S11.** FT-IR spectra of sulfonated LLDPE fibers with a melt flow index of 1.1, 10, and 20 at a sulfonation time of 2.5h under a pressure of 5 bar and temperature of 130°C and draw ratio (a) Sulfonated LLDPE fibers with a melt flow index of 1.1, (b) Sulfonated LLDPE fibers with a melt flow index of 10, (c) Sulfonated LLDPE fibers with a melt flow index of 20.


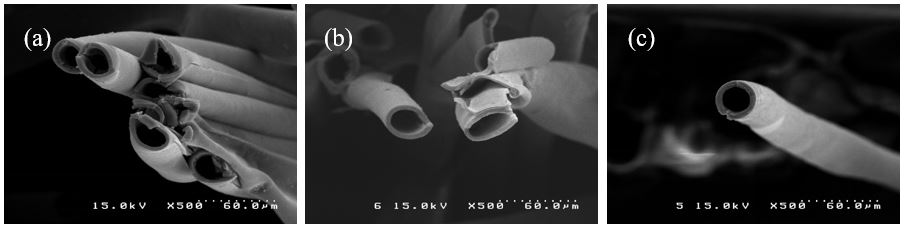


**Figure S12.** Polyethylene-based carbon fibers prepared from partially drawn LLDPE fibers with a sulfonation pressure of 1 bar at 130°C for 2.5h (a) CL1.1, (b) CL10, (c) CL20.

**
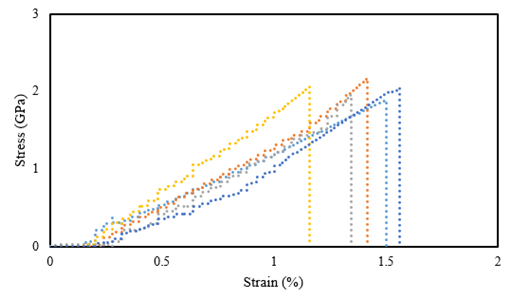
**

**Figure S13.** Stress-strain curves of the polyethylene-based carbon fibers prepared from partially drawn LLDPE fibers.


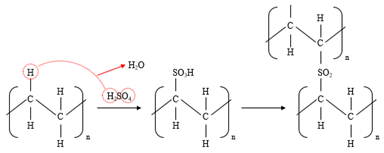


**Figure S14.** Suggested chemical transformation mechanism of polyethylene by sulfuric acid.


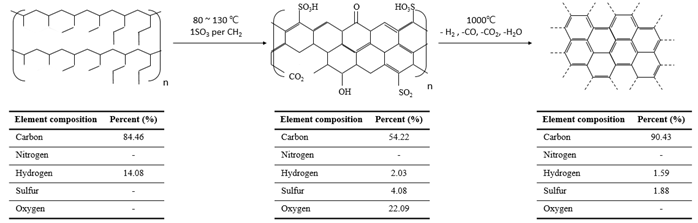


**Figure S15.** Suggested cross-link mechanism and carbonization mechanism of the LLDPE fibers.


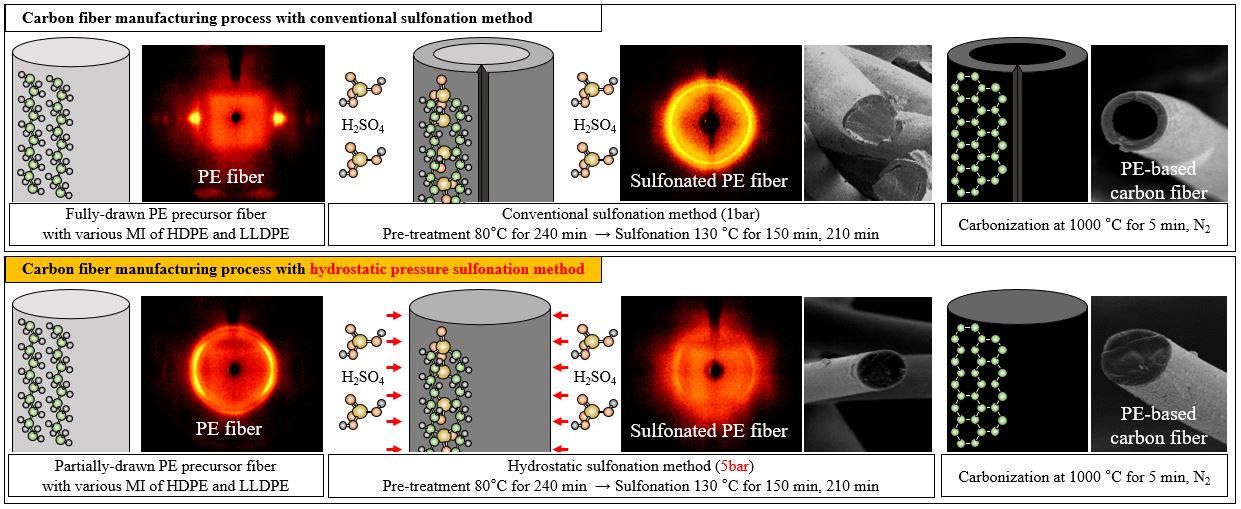


**Figure S16**. Manufacturing process and schematic diagram of polyethylene based carbon fiber through conventional method and hydrostatic pressure sulfonation method.
